# Filarial nematode phenotypic screening cascade to identify compounds with anti-parasitic activity for drug discovery optimization

**DOI:** 10.1016/j.ijpddr.2022.06.002

**Published:** 2022-06-23

**Authors:** Natalie Hawryluk, Li Zhiru, Clotilde Carlow, Suzanne Gokool, Simon Townson, Tamara Kreiss, Agnieszka Chojnowski, Monika Prorok, John Siekierka, Alexandra Ehrens, Marianne Koschel, Nathaly Lhermitte-Vallarino, Coralie Martin, Achim Hoerauf, Geraldine Hernandez, Stacie Canan, Vikram Khetani, Jerome Zeldis, Sabine Specht, Marc P. Hübner, Ivan Scandale

**Affiliations:** a Bristol Myers Squibb, San Diego, CA, USA; b New England Biolabs, Ipswich, MA, USA; c Northwick Park Institute for Medical Research, London, UK; d Sokol Institute of Pharmaceutical Life Sciences, Montclair State University, Montclair, NJ, USA; e Institute for Medical Microbiology, Immunology and Parasitology, University Hospital Bonn, Germany; f German Center for Infection Research (DZIF), Partner Site Bonn-Cologne, Bonn, Germany; g Unité Molécules de Communication et Adaptation des Microorganismes (MCAM, UMR 7245), Muséum national d’Histoire naturelle, Paris, France; h Bristol Myers Squibb, Summit, NJ, USA; i Celgene Global Health, Summit, NJ, USA; j Drugs for Neglected Diseases *Initiative*, Geneva, Switzerland

## Abstract

Filarial diseases, including lymphatic filariasis and onchocerciasis, are considered among the most devastating of all tropical diseases, affecting over 86 million people worldwide. To control and more rapidly eliminate onchocerciasis requires treatments that target the adult stage of the parasite. Drug discovery efforts are challenged by the lack of preclinical animal models using the human-pathogenic filariae, requiring the use of surrogate parasites for *Onchocerca volvulus* for both *ex vivo* and *in vivo* evaluation. Herein, we describe a platform utilizing phenotypic *ex vivo* assays consisting of the free-living nematode *Caenorhabditis elegans*, microfilariae and adult filariae of the bovine filariae *Onchocerca lienalis* and *Onchocerca gutturosa*, respectively, as well as microfilariae and adult filariae of the feline filariae *Brugia pahangi*, the rodent filariae *Litomosoides sigmodontis* and the human-pathogenic filariae *Brugia malayi* to assess activity across various surrogate parasites. Utilization of those surrogate nematodes for phenotypic *ex vivo* assays in order to assess activity across various parasites led to the successful establishment of a screening cascade and identification of multiple compounds with potential macrofilaricidal activity and desirable physicochemical, MW = 200–400 and low lipophilicity, logP <4, and pharmacokinetic properties, rat and human liver S9 stability of ≥70% remaining at 60 min, and AUC exposures above 3 μM h. This platform demonstrated the successful establishment of a screening cascade which resulted in the discovery of potential novel macrofilaricidal compounds for futher drug discovery lead optimization efforts. This screening cascade identified two distinct chemical series wherein one compound produced a significant 68% reduction of adult *Litomosoides sigmodontis* in the mouse model. Successful demonstration of efficacy prompted lead optimization medicinal chemistry efforts for this novel series.

## Highlights

• Successful establishment of a screening cascade for filaricidal drug discovery.
• Chemical series with promising physiochemical and pharmacokinetic properties.
• Identification of a hit compound with macrofilaricidal efficacy.

## Graphical abstract

**Figure:**
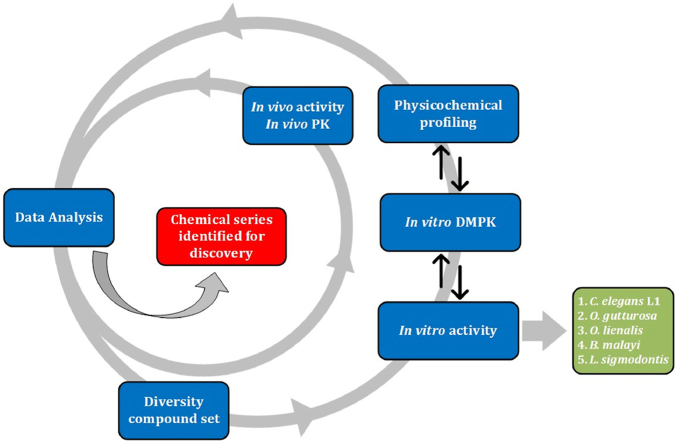

## 1 Introduction

Filarial nematodes are an important and diverse group of pathogens responsible for a number of parasitic diseases, including lymphatic filariasis and onchocerciasis. Due to their high rate of infection and associated severe pathology, filarial diseases are considered among the most devastating of all tropical diseases, affecting over 86 million people worldwide (WHO, 2017a, b). The filarial parasites *Wuchereria bancrofti, Brugia malayi,* and *Brugia timori* all represent serious public health threats as they are the causative agents of lymphatic filariasis or elephantiasis (Townson et al., 2007; Taman and Azab, 2014). Whereas *Onchocerca volvulus* and *Loa loa* are the causative agents of subcutaneous filariasis, with *O. volvulus* infection being the cause of river blindness (Katiyar and Singh, 2011; Hopkins, 2013). While significant progress has been made towards the goal of reducing the prevalence of these diseases, current treatments are targeted at the progeny of worms (microfilariae) and do not kill or permanently sterilize the adult worms, thus only temporarily stopping transmission of the disease. Therefore, repeated treatments for up to 5 years for lymphatic filariasis, and as much as 12–15 years for onchocerciasis are required (Plaisier et al., 1991). Mass drug administration of the microfilaricide ivermectin, which does not kill adult worms, has been used for over 30 years as a single agent for treatment of onchocerciasis in endemic areas (Ashour, 2019). In areas co-endemic for loiasis and onchocerciasis, neurological adverse events consisting of coma, some of which resulted in encephalopathy, Parkinson's disease, or death, have been observed in a limited patient population treated with ivermectin, as a result of rapid depletion of circulating *Loa loa* microfilariae (Holmes, 2013). The need for repeated drug administration, concerns about serious *L. loa*-related adverse events, and the possible emergence of ivermectin-resistant *O. volvulus* have heightened the need for compounds that exhibit adult stage selectivity (macrofilaricidal) or long-lasting sterilizing effects (Hawryluk, 2020). Current efforts to control and eliminate onchocerciasis are hindered by the lack of medicines that target the adult worm stage (Hawryluk and Scandale, 2019). In addition, a major challenge hampering drug discovery is finding suitable preclinical animal models, as *O. volvulus* can only develop fully in humans and primates. This requires the use of surrogate nematodes for both *ex vivo* and *in vivo* evaluation. The free-living nematode, *Caenorhabditis elegans* is a well-established model for high throughput screening commonly used to assess anthelmintic activity of large libraries of compounds (Sepulveda-Crespo et al., 2020). *Brugia* species, such as *B. malayi*, a human pathogen, and the animal filariae *Brugia pahangi* and *Litomosoides sigmodontis*, are the most suitable sources of viable microfilariae and adult worms for high-throughput screening in immunocompetent rodents (Townson et al., 2007). Surrogate parasites for *O. volvulus* include *Onchocerca* species which infect cattle, such as *Onchocerca gutturosa* and *Onchocerca ochengi,* with *O. ochengi* being the closest relative to the human parasite in that it forms nodules that resemble those of *O. volvulus* (Allen et al., 2008).

**Fig. 1:**
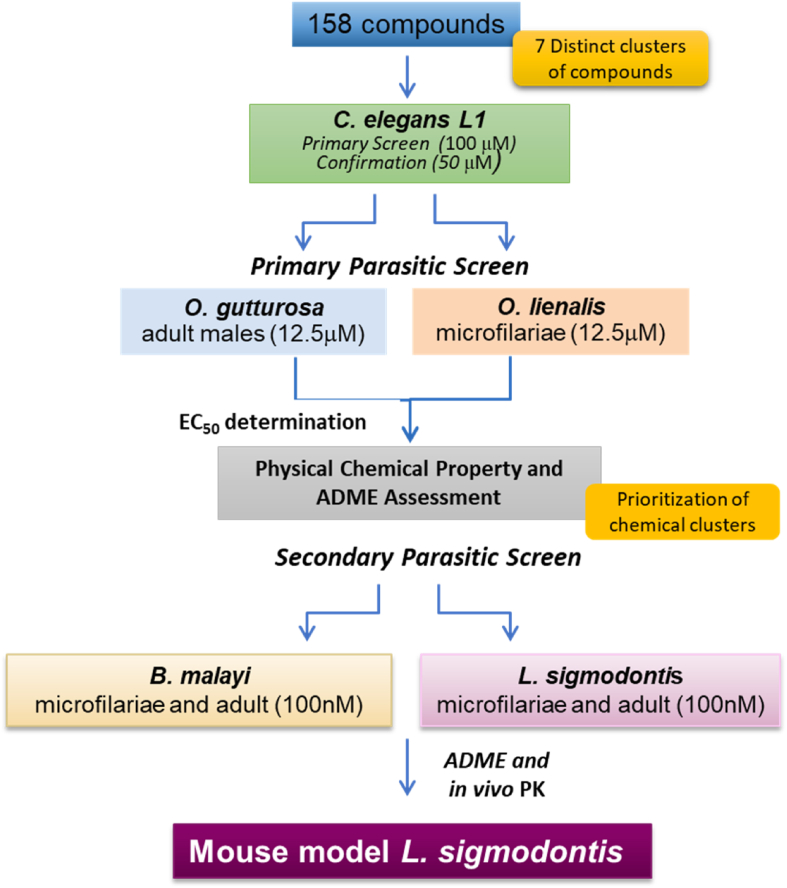
*Ex vivo* compound screening pathway. Compounds representing 7 distinct chemical classes were evaluated in *C. elegans, O. gutturosa,* and *O. lienalis. C. elegans* assay observation period was 3 days–12 days. *O. gutturosa, O. lienalis, B. malayi,* and *L. sigmodontis* motility assays are measured over 5-days. Compounds showing <12 μM against *O. gutturosa* progressed to EC_50_ determination. Activity (EC_50_ values) in the micromolar (μM) range against adult parasites are considered positive hits. “Hit” compounds (<12 μM) were assessed and prioritized based on physical chemical properties and ADME. Secondary assays were performed on select compounds in *B. malayi,* and *L. sigmodontis* microfilariae and adult female filariae at 100 nM. Selected compounds were evaluated for oral exposure *in vivo* PK and were evaluated in mice that harboured adult worms of the rodent filarial nematode *L. sigmodontis*.

For filarial indications, there are few identified targets allowing the establishment of target-based assays. Whole organism cross-screening assays have therefore been utilized to identify compounds across various chemical series with anti-parasitic activity (Fig. 1). Available phenotypic assays have relatively low throughput; therefore, ranking, and triaging molecules prior to characterization through these different assays is essential. Utilizing the heritage Celgene Global Health collection enriched with chemical matter in the neglected disease (NTD) space, compounds were initially identified by their ability to either kill or interrupt the developmental stages of *C. elegans* (Subbian et al., 2016; Mundhra et al., 2018; Tummalapalli et al., 2018; Akao et al., 2021*)*. Primary parasitic evaluation showed reduction of motility in *Onchocerca* nematodes, *O. gutturosa* and *O. lienalis*. Prioritization of chemical classed based on *Onchocerca* activity, physiochemical and pharmacokinetic properties was established prior to subsequent evaluation in secondary parasitic filarial nematodes, *B. malayi,* and *L. sigmodontis*. Lastly, for *in vivo* proof of concept evaluation, *in vivo* pharmacokinetic (PK) properties of the compounds were further evaluated. Thus, the utilization of phenotypic *ex vivo* assays in order to assess activity across various parasites and ultimately the identification of novel compounds against filarial diseases will be discussed.

## 2 Materials and methods

### 2.1 *Caenorhabditis elegans*: *ex vivo* screening model

Synchronized L1 larvae were hatched from bleached eggs and were grown in 100 μL S medium with *E.coli* (dead) supplied as a food source in a 96-well plate (Zhang and Kuhn, 2013). Ten L1 larvae were distributed into each well of the 96-well plate. Compounds were initially tested at 100 μM and 50 μM in triplicate. Worms were incubated at 20 °C for up to 12 days. Food consumption of dead *E.coli* was measured daily using a spectrometer reading at OD600nm and used as an indicator for worm health and viability (Win et al., 2013). In general, the L1 stage develops into the adult stage within 3 days. The observation period was extended beyond 3 days–12 days to capture the full impact of compounds. Worm development was examined microscopically daily using a standard dissecting microscope (Motic K series) and the worm condition (live/dead) was reported on day 7 of the culture. Viability was scored based on food consumption (Gomez-Amaro et al., 2015) motility and morphology. A change in food consumption between day 1 and day 7 below ∼0.3 indicates a compound effect. Larval stage determination and development was assessed based on size and morphology as described (Wood, 1988). One generation of worm development was followed for growth defects/arrest.

### 2.2 Onchocerciasis: *ex vivo* screening assays with *O. gutturosa* adult worm and *O. lienalis* microfilariae

*O. gutturosa* adult male worms were obtained postmortem from freshly slaughtered cattle. The worms were dissected from the nuchal ligament connective tissues of naturally infected cattle in Gambia, West Africa; The West Africa Livestock Innovation Centre (WALIC), Banjul, purchased material from local butchers for use in this study.

A single large batch of *O. lienalis* microfilariae was obtained post mortem from the peri-umbilical skin area of freshly slaughtered, naturally infected cattle in the UK following the procedure described by (Tagboto and Townson, 1991) and the references therein. The extracted microfilariae were cryopreserved using a 2-step incubation technique with ethanediol as a cryoprotectant (Ham et al., 1981), stored in liquid nitrogen and thawed when required for immediate use.

Using a dissecting microscope and micropipette, microfilariae were then transferred in groups of 5 into each well of a 96-well plate (Fisher Scientific, UK). The adult *O. gutturosa* worms were transferred individually to each well of a sterile 24-well (2 mL) plate (Fisher Scientific, UK) and maintained for at least 24 h in culture before use. For the *O. lienalis* 5-day motility assay, a confluent layer of a monkey kidney cell line (LLCMK2, ECACC, UK) was used as feeder cells. Both *O. gutturosa* adult worm and *O. lienalis* microfilariae cultures were maintained in Minimum Essential Medium with Earl's Salts and L-glutamine (Life Technologies Ltd, UK) supplemented with 10% heat-inactivated newborn calf serum (Life Technologies Ltd, UK) and antibiotics/antimycotics (200 units/mL penicillin, 200 μg/mL streptomycin and 0.5 μg/mL amphotericin B; Life Technologies Ltd, UK).

Only normally active worms were used for the test and all assays were conducted at 37 °C and 5% CO_2_. The positive control drug used was Immiticide (melarsomine dihydrochloride, Merial) supplied as a dry solid. Known amounts of this compound were solubilized in 1 mL dimethylsulphoxide (DMSO) and the stock solution was allowed to stand at room temperature prior to use. Test compounds were screened at a top concentration of 12.5 μM followed by serial 1 in 4 drug dilutions to estimate the EC_50_ values, with the positive control at concentrations of 12.5 μM, 3.13 μM, 0.78 μM, and 0.195 μM. The test compounds (2 *O. gutturosa* adult worms/5 *O. lienalis* microfilariae in 1 well per drug concentration) were compared to untreated controls (6 *O. gutturosa* adult worms/5 *O. lienalis* microfilariae in each of 6 wells) and the positive control (2 *O. gutturosa* adult worms/5 microfilariae in 1 well per drug concentration). The final DMSO solvent concentration was 0.25% both at the top concentration of 12.5 μM and for the untreated control groups. The activity levels of *O. gutturosa* adult worms were assessed by the measurement of mean motility scores on a scale of 0 (immotile) to 10 (maximum) every 24 h, terminating at 120 h, using an Olympus inverted microscope. For *O. lienalis* microfilariae, the motility of the microfilariae was classified using an Olympus inverted microscope as normal (continuous rapid sinuous movement, scored as 3), marginally impaired (slower than normal movement, scored as 2), severely impaired (scored as 1), or immotile (scored as 0) after 120 h of drug exposure. The motility reduction was produced by comparison of the motility levels of the drug treated group to the negative control groups.

The EC_50_ values were calculated using a sigmoidal interpolation in Graph Pad Prisme 7 data analysis software. The test drug is considered active when a motility reduction of ≥50% is observed by comparison to the untreated controls. In addition, biochemical evaluation of *O. gutturosa* adult worm viability was assessed using MTT/formazan colorimetry. The MTT assay was carried out after the last motility reading (120 h). Single intact worms were placed in each well of a 48-well plate (Fisher Scientific, UK) containing 0.5 mL of 0.5 mg/mL MTT (Sigma, UK) in PBS solution, and then incubated for 30 min at 37 °C. The worms were removed, blotted carefully, and individually transferred to separate wells of a 96-well microtiter plate, each containing 200 μL of DMSO to solubilize the formazan. After 1 h the plate was gently agitated to disperse the color evenly and the absorbance value (optical density, OD) of the resulting formazan solution was determined at 490 nm using an absorbance microplate reader (Biotek ELx800, Fisher Scientific, UK). The results are expressed as the mean OD per drug concentration. Inhibition of formazan formation is correlated with worm damage or death.

### 2.3 Cytotoxicity assay with monkey kidney cell feeder layer

The monkey kidney cell feeder layer (LLCMK2, ECACC, UK) used for the *O. lienalis* microfilariae assays was also used to assess the cytotoxicity of candidates. The cell cultures are sub-passaged every 15–20 days using trypsin to detach the cells and split in a 1:4 ratio. All cultures and assays are conducted at 37 °C under an atmosphere of 5% CO2 in air. The cell feeder layer was examined by light microscopy (CETI (Belgium) inverted microscope) to look for acute toxicity caused by drug exposure. Compounds were tested in duplicate at 12.500, 3.100, 0.780 and 0.190 μM for each compound. Observations were classified as i) cell monolayer normal in appearance, equivalent to (no drug) control. ii) cells with some abnormal appearance, typically including mis-shapen cells, possible thinning of the monolayer or patchy areas, but with the majority of the cells remaining attached to the bottom of the plate. iii) toxic to cells, showing acute toxicity, detachment of the monolayer from the bottom of the plate and/or individual cells rounded-up and large-scale cellular debris observed. Compounds found to be toxic to the cell feeder layer may be re-tested at lower concentrations to see if there is selective activity against the parasite (and not the cells).

### 2.4 *Ex vivo* motility assays with *B. malayi, B. pahangi* and *L. sigmodontis*

Adult and microfilarial *B. malayi* and *B. pahangi* parasites, harvested from infected jirds, were procured from the NIAID/NIH Filariasis Research Reagent Resource Center (FR3). Adult and microfilariae of *L. sigmodontis* were procured from TRS labs Inc. (Athens, GA). Adult worms were plated in 24-well plates with 2 mL of Advanced RPMI 1640 medium (Invitrogen) supplemented with 25 mM HEPES, 2 mM L-glutamine (Invitrogen), 100 U/mL penicillin (Invitrogen), 100 mg/mL streptomycin (Invitrogen), 2.5 mg/mL amphotericin B solution (Invitrogen), and 5% heat inactivated fetal bovine serum and placed in a 37 °C humidified incubator with 5% CO_2_. After 24 h, adult worms were selected based upon motility as described (Patel et al., 2011). After scoring for motility, 4–6 highly motile worms were selected for each treatment group and were transferred to new plates. Microfilariae were centrifuged at 5000×*g* for 5 min, and re-suspended in 2 mL of media. Microfilarial density was determined using a hemocytometer and were plated in a 96-well plate at 80 microfilariae/well with 200 μL of complete media. Treatment groups received compounds (0.1% DMSO) at 1 μM and 100 nM with 0.1% DMSO as a vehicle control. Cultures were incubated at 37 °C in a humidified incubator with 5% CO_2_ (Townson et al., 1987). Worms were transferred into a new plate containing fresh media and drug every 48 h. Parasite and microfilariae motility were given a score from 0 to 4 with 4, rapid movement and largely coiled; 3, moderated movement and uncoiled; 2, slow movement and uncoiled; 1, twitching movement and uncoiled; 0, no motility (dead).

### 2.5 Ethics statement

All experimental procedures were performed in accordance with EU directive 2010/63/EU and the relevant national legislation, the “Décret no 2013–118, 1er février 2013, Ministère de l’Agriculture, de l’Agroalimentaire et de la Foret”, national license number 75–1415. Animal protocols were approved by the ethical committee of the MNHN (Comité Cuvier, License: 68–002) and by the “Direction départementale de la cohésion sociale et de la protection des populations” (DDCSPP) (No. C75-05-15). The animal experiments done at IMMIP were approved by the Landesamt für Natur, Umwelt und Verbraucherschutz, Cologne, Germany (AZ 84-02.04.2015.A507). All pharmacokinetic experiments were approved by the Institutional Animal Care and Use Committee (IACUC) of Bristol Myers Squibb.

### 2.6 *In vivo* analysis with *L. sigmodontis*

The life cycle of the filarial nematode *L. sigmodontis* was maintained in the animal facilities at the MNHN and at the IMMIP in jirds (*Meriones unguiculatus*) and cotton rats (*Sigmodon hispidus*), respectively. Host animals used for infections at the MNHN were female BALB/c mice purchased from Envigo, France. Host animals used for infections at the IMMIP were female BALB/c mice obtained from Janvier (Saint-Berthevin, France). All animals were kept on a 12 h light dark cycle and were infected between 6 and 8 weeks of age. Only female animals were used in this study to avoid sex-based differences, as female BALB/c mice are more susceptible for the infection (Petit et al., 1992). At MNHN, female BALB/c mice were inoculated with 40 L3 in 200 μL of RPMI 1640 by subcuteaneous injection in the left lumbar area. Infective L3 were recovered from the vector, the tropical rat mite *Ornithonyssus bacoti*, as previously described (Karadjian et al., 2017). At IMMIP, female BALB/c mice were infected naturally via the exposure to *L. sigmodontis*-infected *O. bacoti* (Frohberger et al., 2020)*.* Treatments were initiated at 33–35 days post-infection (dpi) in mice. 5–6 animals were used for oral treatment of candidates, additional groups included orally treated vehicle controls (n = 6–7 animals) and as positive control flubendazole administered subcutaneously with 2 mg/kg once per day for five days (n = 3–5 animals). Single experiments were performed. Doses in mg of drug substance per kg of body weight of animals are indicated in the text. Mice were sacrificed at 75–78 dpi. The mice were anesthetized with 1.6 mg Chlorhydrate d'oxybuprocaine (lidocaine) then sacrificed by bleeding (at MNHN) or exposure to an overdose of isoflurane (IMMIP), the pleural cavity was washed 10 times with 1 mL of cold phosphate buffered saline (PBS) to collect filariae as previously described (Karadjian et al., 2017). The remaining worms were isolated using forceps. The recovered *L.* *sigmodontis* adult worms were counted and analyzed by light microscopy to identify males and females.

### 2.7 Mouse pharmacokinetics

All studies were conducted in fed animals (male CD-1 mice) by Charles River Laboratories (Wilmington) INC. Oral dosing (at 30 mg/kg, 10 mL/kg) was as a suspension in 0.5% carboxymethylcellulose and 0.25% Tween 80 in water. Plasma samples (at 0.5, 1.5, 3, 5, 8 h) were analyzed by LC−MS/MS, and the PK parameters were calculated using Phoenix WinNonlin software.

### 2.8 Liver S9 metabolic stability assay

Incubations were performed at 37 °C in a Dubnoff Shaking water bath using 2 mL 96-well incubation plates. Rat and human S9 protein concentrations were 0.75 mg/mL and 1.2 mg/mL, respectively. The final concentrations of NADPH, UDPGA, and GSH were 1, 0.5, and 2.5 mM, and the final concentration of PAPS was 0.05 mg/mL. Substrate concentrations were 3 μM, incubation time was 60 min, and all tests were done in triplicate. The incubation was conducted in 200 mM Tris buffer containing 2 mM magnesium chloride, pH = 7.4 and the total incubation volume was 0.5 mL. Samples were analyzed via LC-MS/MS and reported as percentage remaining after incubation for 60 min.

### 2.9 Chemical properties

Calculations (MW, TPSA, LogP, LE, LLE) were carried out and plotted using Dotmatics Vortex v2016.10.56814.

## 3 Results

### 3.1 Primary screening against *C. elegans* identifies actives across multiple chemical series

**Fig. 2:**
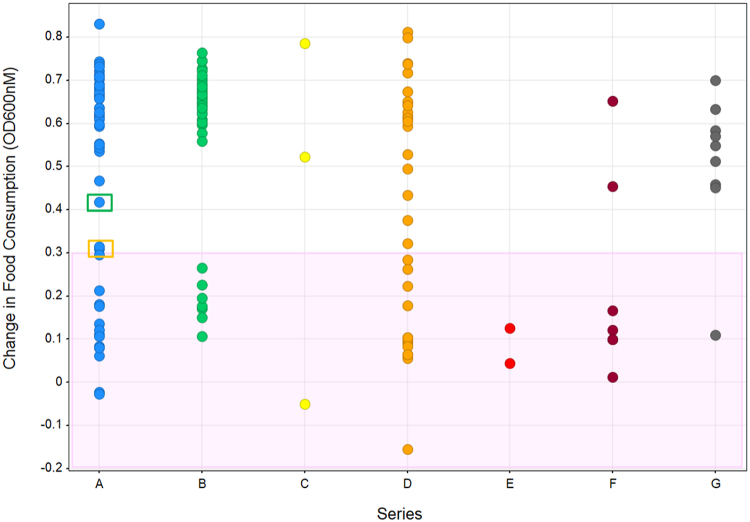
*C. elegans* assessment of compound viability based on food consumption. Compounds were tested at 50 μM on L1 larvae of *C. elegans* in 96-well plates. Each well contained 100 μL of S medium, 10 L1 larvae, 1 μL of compound dissolved in DMSO or DMSO control, and dead *E. coli*. OD600nm was measured on day 1 and day 7 to measure the amount of *E. coli* inside the well. The difference in OD600nm between day 1 (*E. coli* added) and day 7 (*E. coli* remaining) was plotted. Compound 1 is indicated by the green box, Compound 2 is indicated by the orange box. Single experiment with 10 larvae per condition. (For interpretation of the references to color in this figure legend, the reader is referred to the Web version of this article.)

A targeted set of 158 diverse compounds from the heritage Celgene Global Health (CGH) collection, consisting of 7 distinct structural classes, Series A-G, was initially screened against the L1 developmental stage of *C. elegans* at 100 μM concentration followed by confirmation testing at lower concentration of 50 μM. During testing, two main parameters were examined at day 7: food consumption, an indicator of worm health/viability, and microscopic examination of phenotypic development and viability. A third of the compounds when tested at 50 μM were shown to reduce food consumption, which is indicative of compromised worm viability (a change in food consumption below ∼0.3) Fig. 2, area shaded in pink). Some compounds caused worm death and many compounds resulted in developmental arrest at a larval stage (L1-L4) or of young adults. In some cases, the adult stage was reached but the worms were sterile, failing to produce f1 progeny (SI Table 2 for phenotypic data).

### 3.2 Screening confirms activity against *Onchocerca lienalis* and *Onchocerca gutturosa*

**Fig. 3:**
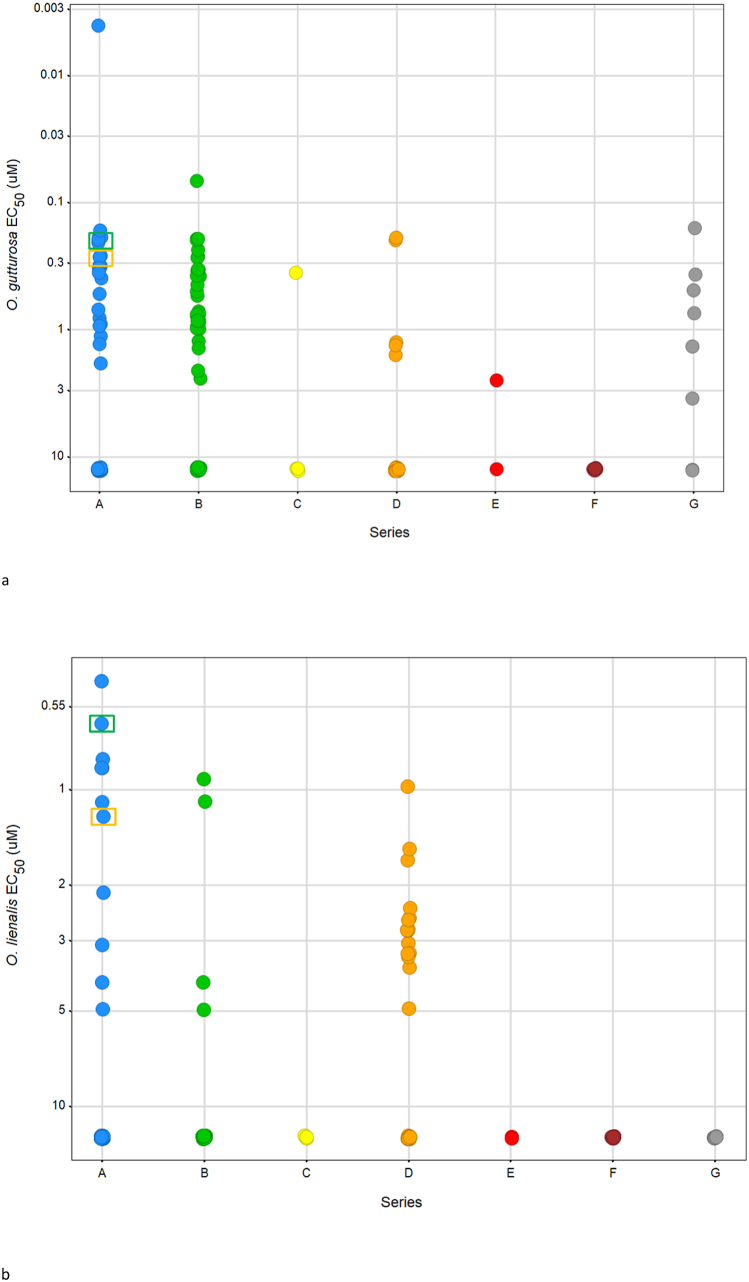
*Ex vivo* screening against adult *O. gutturosa* and *O. lienalis* microfilariae. 3a Circles represent the motility inhibition EC_50_ (μM) of *O. gutturosa* adult worms after 5 days of *in vitro* culture with test compounds. Compounds were tested at a top concentration of 12.5 μM and observed for reduction in motility as represented by the EC_50_ (μM). **3b** Circles represent the motility inhibition EC_50_ (μM) of *O. lienalis* microfilariae after 5 days of *in vitro* culture with test compounds. Compound 1 is indicated by the green box, Compound 2 is indicated by the orange box. Single experiment with 2 adult *O. gutturosa* filariae or 5 *O. lienalis* microfilariae per test compound condition. (For interpretation of the references to color in this figure legend, the reader is referred to the Web version of this article.)

As onchocerciasis was the primary goal, the set of 158 compounds were tested against the adult stage of *O. gutturosa* and microfilariae of *O. lienalis.* Reduction of motility and viability was observed across select compounds in series A, B, D, and G against the adult stage *O. gutturosa* (Fig. 3a). From the set 49 compounds indicating a >70% reduction in food consumption in *C. elegans* 29 compounds exhibited activity against the adult stage *O. gutturosa* parasites *ex vivo.* From the entire initial 158 set, 67 compounds had activity against the adult stage of *O. gutturosa* and a lack of activity against the *O. lienalis* microfilariae, EC_50_ > 12.5 μM (majority of the circles residing below >10 μM in Fig. 3b). Only 31 compounds from the 158 compound set demonstrated modest activity, EC_50_ > 5 μM, against the *O. lienalis* microfilariae, (Fig. 3b). Just 5 compounds, all residing in Series A, had activity with EC_50_ < 1.0 μM for both adult *O. gutturosa* and *O. lienalis* microfilariae (SI Table 1 for complete EC_50_ data). Notably, compounds showing reduction of motility in adult *O. gutturosa*, EC_50_ < 1.0 μM, also impacted the worm viability at 12.5 μM as demonstrated using MTT/formazan colorimetry (SI Fig. 4). Percentage reduction (compared to the untreated control) of formazan formation was correlated with worm damage or death. The positive control, immiticide, has an *O. gutturosa* EC_50_ = 0.59 μM with MTT reduction of 96.3% at 12.5 μM (SI Fig. 1).

**Table 1:** Mouse pharmacokinetic parameters following oral administration.

Mouse Pharmacokinetic (PO) Parameters in Mice^a^^,^^b^						
	Compound 1			Compound 2		
C_max_ (μM)^c^	1.00	±	0.563	0.697	±	0.180
T_max_ (hr)^d^	0.50	±	0.00	0.50	±	0.00
AUC_(0-8)_ (μM·h)^e^	1.13	±	0.48	1.58	±	0.27

a Male CD-1 mice (n = 4).

b Dose: 30 mg/kg.

c Maximum concentration: C_max_.

d time at maximum concentration T_max_.

e Area under the curve from 0 to 8 h AUC_(0-8)_.

**Fig. 4:**
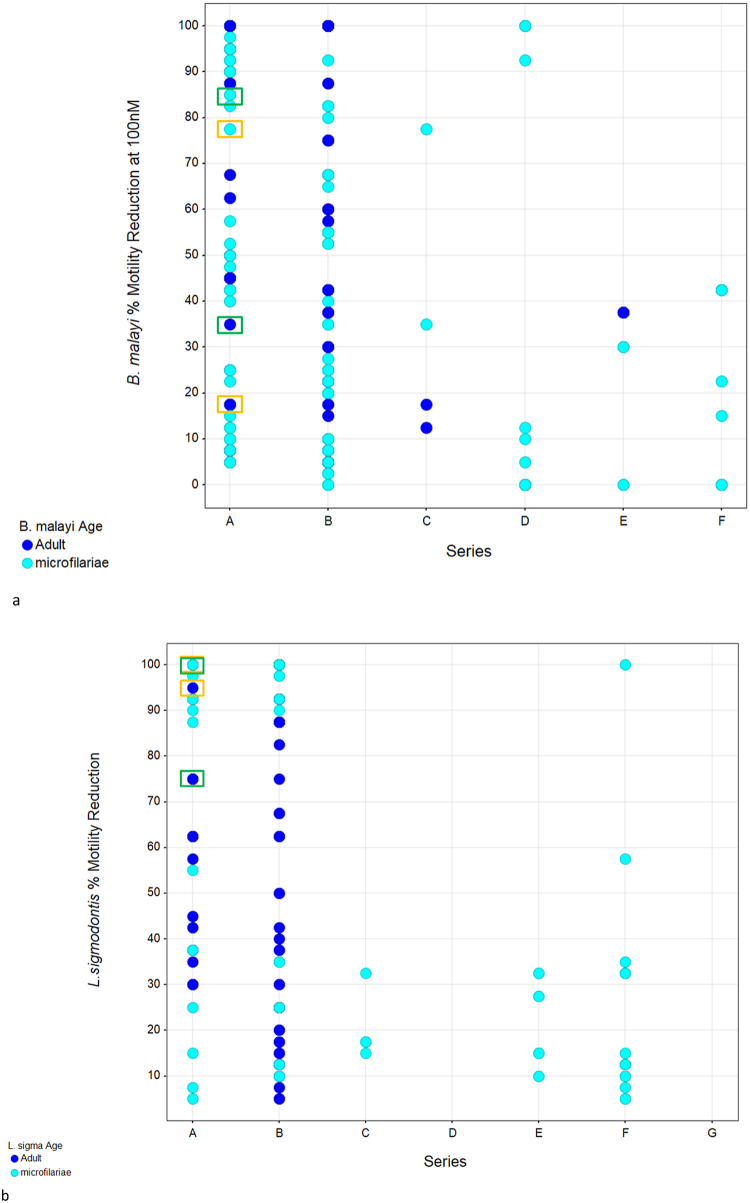
*Ex vivo B. malayi* and *L. sigmodontis* screening. All compounds were screened at 100 nM and measured for reduction in overall motility. Shown are motility scores after 5 days of culture converted to a percentage reduction where a score of 4 (rapid movement and largely coiled) = 0% motility reduction and score of 0 (no motility, dead) = 100% motility reduction. Dark blue circles indicate adult worms and light blue circles indicate microfilariae. Compound 1 is indicated by the green box, Compound 2 is indicated by the orange box. Single experiment with 4–6 adult *L. sigmodontis/B. malayi* filariae or ∼80 *L. sigmodontis/B. malayi* microfilariae per condition. (For interpretation of the references to color in this figure legend, the reader is referred to the Web version of this article.)

### 3.3 Secondary screening confirms activity against *Brugia malayi* and *Litomosoides sigmodontis*

For evaluation against additional parasitic filarial nematodes, selected compounds from the 158 compound set were tested at a single concentration, 100 nM, and evaluated based on the inhibition of movement and motility of microfilariae and adult worms of *B. malayi* and *L. sigmodontis* every 24 h over 5 days. By day 5, series A and B compounds showed a range of activity against microfilariae and adult worms of both *B. malayi* and *L. sigmodontis*, while series D, E, and F showed modest to no impact against either parasite (Fig. 4 and SITable 1 for data). Compounds which showed activity against *L. sigmodontis* and *B. malayi* typically also showed activity against *O. gutturosa*.

### 3.4 Assessment of physical chemical properties pharmacokintetic parameters

**Fig. 5:**
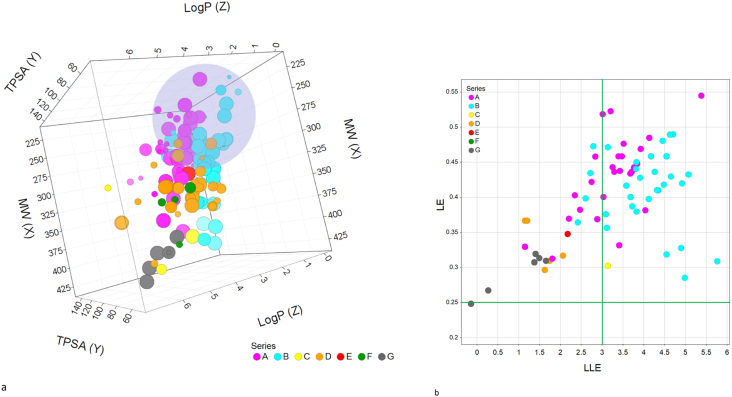
Analysis of chemical properties of series A-G. 5a Compounds were analyzed based upon their TPSA (total polar surface area), LogP, MW (molecular weight), **5b** LE (ligand efficiency), and LLE (ligand lipophilicity efficiency), sized by reduction of *O. gutturosa* motilty (single experiment done in triplicates) and colored by chemical series.

**Fig. 6:**
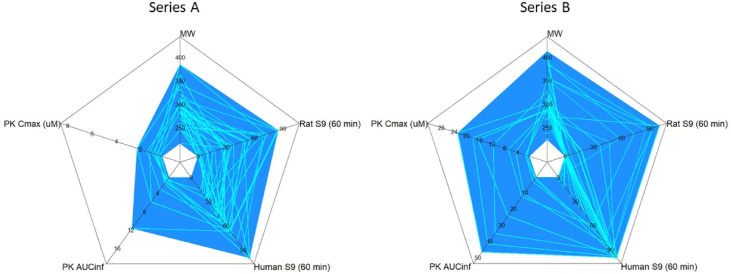
Analysis of ADME Properties of Series A and B. ADME properties were evaluated for compounds based upon MW (molecular weight), phamacokintetic parameters (PK): maximum concentration (C_max_ (μM)), area under the curve at infinite time (AUC_inf_, (μM.h)), human and rat liver microsomal stability, and S9 fraction (percentage remaining at 60 min). Data from a single experiment done with four mice per group.

Compounds across all the series were evaluated for physical chemical and pharmacokintetic properties. Ideal physical chemical properties are molecular weight (MW) within a range of 200–400, low lipophilicity, logP <4, and a total polar surface area (TPSA) < 100. Compounds in series A and B with *O. gutturosa* EC_50_ < 1 μM have favorable physical chemical properties with the majority of the compounds having a MW within a range of 200–400, logP <4, and TPSA <100 (Fig. 5a). Compounds falling with ligand efficiency (LE) = 0.25–0.35 and ligand lipophilicity efficiency (LLE) = 3–5 were considered to be good hit starting points, whereas LE = 0.35–0.45 and LLE = 5–7 (upper right quadrant, Fig. 5b) are more consistent with highly optimized development compounds (Hopkins et al., 2004, 2014). The two distinct chemical series, A (colored purple) and B (colored aqua), emerged in desirable chemical space with ligand efficiency LE > 0.35 and ligand lipophilicity efficiency LLE >3 (Fig. 5b). Unfortunately compounds in series C, D and G with MW nearing 400, logP ∼4 and LE in an undesirable space (Fig. 5a and b) precluded them from further evaluation (SI Table 3 for complete data). Both series A and B have desirable ADME properties, absorption, distribution, metabolism, and excretion (ADME). Rat and human liver S9 stability of ≥70% remaining at 60 min (Duffus et al., 2007), and moderate oral rodent pharmacokinetics (PK), AUC exposures above 3 μM h (Fig. 6). Upon assessment of rat and human liver S9 stability, select compounds from series A and B were evaluated in mouse pharmacokinetic studies. Following oral administration in mice, compounds 1 and 2 showed sufficient plasma exposure when dosed 3 times a day (TID) to progress into the efficacy studies (Table 1).

**Table 2:** Compound analysis in the *L. sigmodontis* murine model of filariasis.

Compound	Number of mice	Dose	Worm number Median ± Range (Standard error of means)	Reduction Adult Worms^a^
Vehicle^b^ (0.5% CMC/0.25% Tween 80 in Millipore water)	7	TID, PO 5 days	9 ± 25 (3.82)	–
Vehicle^c^ (0.5% CMC/0.25% Tween 80 in Millipore water)	6	TID, PO 5 days	6.5 ± 9 (1.45)	–
Flubendazole^b^	5	2 mg/kg, QD SC, 5 days	0 ± 1 (0.20)	98.4% (P = 0.024)
Compound 1 (structure not released)	6	3 × 30 mg/kg TID, PO 5 days	9.5 ± 14 (2.35)	14.2% (P = 0.714)
Flubendazole^c^	3	2 mg/kg, QD SC, 5 days	0 ± 0 (0.00)	100% (P = 0.007)
Compound 2	5	3 × 30 mg/kg TID, PO 5 days	1 ± 4 (0.77)	68.4% (p = 0.032)

a Percentage reduction in adult *L. sigmodontis* was measured in mice treated orally three times per day with 30 mg/kg of compound 2 (0.5% CMC/0.25% Tween 80 in Millipore water) for 5 days, and flubenzadole (2 mg/kg, subcutaneously in 0.1% Tween 80 in 0.5% HEC once-daily for 5 days). Percentage reduction of total worm count was determined from the mean of treatment groups and compared to mean of vehicle controls. Analysis was done using unpaired 2 tailed *t*-test.

b Vehicle and positive control for compound 1.

c Vehicle and positive control for compound 2. Single experiment with the indicated number of animals per group.

**Table 3:** Compounds 1 and 2 *ex vivo* data summary.

*ex vivo* Assays		
Assay	**Compound 1**	**Compound 2**
*C. elegans* change in food consumption at 100 μM	0.4	0.3
*O. gutturosa* MTT % reduction at 12.5 μM	73.9%	84.7%
*O. gutturosa* EC_50_ (μM)	0.2	0.2
*O. lienali*s EC_50_ (μM)	0.6	1.2
*L. sigmodontis* % adult worm motility reduction at 100 nM	75%	95%
*L. sigmodontis* % mf motility reduction at 100 nM	100%	100%
*B. malayi* % adult worm motility reduction at 100 nM	35%	18%
*B. malayi* % mf motility reduction at 100 nM	85%	77%
Cytotoxicity a (μM)	>12.5	>12.5

a Mammalian cells – monkey kidney cell feeder layer (LLCMK2, ECACC, UK); mf = microfilariae.

### 3.5 *In vivo* screening against *Litomosoides sigmodontis* identifies compound demonstrating reduction of adult worm burden

To investigate *in vivo* efficacy, Compounds 1 and 2 were administered by oral gavage in mice that harboured adult worms of the rodent filarial nematode *L. sigmodontis* (Morris et al., 2013) (Hubner et al., 2009). Groups of 5–7 mice were infected with *L. sigmodontis* infective L3 larvae and treated orally 30 days post infection at 30 mg/kg TID for 5 days. Adult parasites were counted 75–78 days post treatment. While Compound 1 failed to induce a significant reduction in adult worm burden (14.2% reduction), Compound 2 induced a significant reduction of 68% of adult *L. sigmodontis* compared to the vehicle treated control mice (Table 2). *In vivo* efficacy experiment using the *L. sigmodontis* mouse model were performed in two independent laboratories, the University Hospital Bonn and the Muséum Nationale d’Histoire Naturelle (SI Fig. 5).

## 4 Discussion

A targeted set of 158 diverse compounds selected from the heritage Celgene Global Health (CGH) collection was screened against the L1 developmental stage of *C. elegans* at an initial concentration of 100 μM. From this initial phenotypic screen in *C. elegans*, multiple chemical series (A, B, D, and G) identified in total 49 compounds that impacted viability. To assess activity against an *Onchocerca* species, the same set of 158 compounds were tested at 12.5 μM in an *ex vivo* motility assay against *O. lienalis* microfilariae and *O. gutturosa* adult worms and showed correlation with 29 out of the 49 the *C. elegans* hits. From the initial 158 compounds, 67 compounds had specific activity against the adult stage of *O. gutturosa*, as demonstrated by the lack of activity against the *O. lienalis* microfilariae. Select compounds were further cross-screened against other filarial parasites infecting humans to investigate the potential for a broader use across filarial diseases. The compounds were tested against other filarial parasites responsible for filarial diseases in *ex vivo* motility assays, *B. malayi* and the rodent filaria *L. sigmodontis* microfilariae and adults at a single concentration, 100 nM. Compounds which showed activity against *L. sigmodontis* and *B. malayi* correlated well with activity against *O. gutturosa* (SI Table 1 for data).

The majority of the compounds had favorable physical chemical properties with molecular weight (MW), logP, and a total polar surface area all falling within a desirable range, MW = 200–400, logP <4, and TPSA <100. We also adapted the use of efficiency measures to the *O. gutturosa ex vivo* data generated for these series. Ligand efficiency (LE, the measure of potency per atom) and ligand lipophilicity efficiency (LLE, the measure of potency relative to lipophilicity) were assessed to determine the relative efficiency for the various series based on *ex vivo* data. Based on evaluation of these parameters, two distinct chemical series, A and B, emerged in desirable chemical space and LE > 0.35 and ligand LLE >3 (Fig. 5). Assessment of the filarial parasite screening results and evaluation of the drug-like properties of the clustered compound series was based on overall *ex-vivo* potency and compound properties. Compounds in series A and B that showed activity across the multiple parasites were further progressed into ADME pharmacokinetic (PK) assays in order to assess pharmacokinetic properties such as stability, absorption, distribution, metabolism, and excretion (ADME) and *in vivo* viability. In rats, compounds with rat S9 stability of ≥70% at 60 min and a plasma clearance of ≤43 mL/min/kg had the greatest chance of achieving oral exposures above 3 μM h (Kulkarni et al., 2014). Based on the favorable ADME and PK properties, compounds were advanced into *in vivo* PK studies. Due to the unmet need of new treaments that target adult parasites, we chose to further examine the 67 compounds which reduced the motility of *O. gutturosa* adult worms preferentially. To confirm the anthelmintic activity, compounds were prioritized for *in vivo* evaluation based on the following criteria: *O. gutturosa* EC_50_ < 500 nM, selective activity against *O. gutturosa* adult parasites versus *O. linealis* microfilariae (inactive at 12.5 μM, SI > 100), and suitable physicochemical and ADME profiles, logP <4 and rat S9 stability of ≥70% at 60 min with acceptable oral PK. Upon evaluation, 32 compounds had *O. gutturosa* EC_50_ < 500 nM, however after further assessment of both *ex vivo* ADME and *in vivo* pharmacokinetic data, only 2 compounds from series A (Table 3) were progressed into filarial efficacy models based on suitable plasma exposures above *O. gutturosa* EC_50_. *In vivo* proof of principle (POP) is established in mice infected with *L. sigmodontis* with adult parasite and microfilariae assessment post infection. This model is regarded as a reasonable predictor of clinical efficacy (Hubner et al., 2019) (Hubner et al., 2020) and was conducted with compounds with acceptable oral PK in the relevant host species. Therefore, a dosing regimen was chosen to achieve plasma drug concentrations that remained continuously above EC_50_ throughout dosing. Of the two compounds tested, it was hypothesized that only Compound 2, hadachievedsufficient plasma exposure as it showed a significant reduction, 68%, of adult *L. sigmodontis* when treated at 30 mg/kg TID for 5 days, orally, relative to the vehicle control group. Compound 1, although having similar oral PK with a slightly reduced AUC (Table 1) showed no significant reduction of adult *L. sigmodontis* worm burden (9.5 ± 14 worms observed). While not as effective as the standard, subcutaneously (SC) administered flubendazole, this was an encouraging demonstration of POP for this series. The screening paradigm described herein was not aimed to attain all molecules with anthelmintic activity. For instance, one possible limitation of the reported screening cascade is that known anti-helminthic agents such as ivermectin or diethylcarbamazine may have not been identified based on their modest *in vitro* activity against these filarial nematode species in this screen. Nonetheless, employing the *C. elegans* screen we intended to exploit the biological processes which are shared by all nematodes such as molting and reproduction and thus screen relatively large libraries of compounds. The activity of identified hits were subsequently analyzed in various phenotypic screens against different filarial nematode species achieving EC_50_ ≤ 500 nM with desirable physical chemical and pharmacokinetic properties and achieved *in vivo* POP in the *L. sigmodontis* murine model. In summary, we present the establishment of a screening cascade to evaluate potential macrofilarcidal candidates from different chemical series based on *in vitro* and *in vivo* data generated with surrogate filarial species with secondary profiling for physicochemical and pharmacokinetic properties.

## 5 Conclusion

*C. elegans* has been used as a primary screen for drugs including antihelmintics for decades (Elfawal et al., 2019; Hahnel et al., 2020; O'Reilly et al., 2014) allowing for the ease of collecting sufficient data and with low cost and high-through put. Utilizing *C. elegans* and subsequent filarial nematode whole organism cross-screening, we were able to identify compounds from multiple chemical series with broad anti-filarial activity. This platform demonstrated the successful establishment of a screening cascade with the ability to rank and triage compounds prior to evaluation into slow and medium throughput *ex-vivo* assays, and ultimately into *in vivo* disease models. This screening cascade resulted in the identification of potential novel macrofilaricidal compounds for futher drug discovery lead optimization efforts. Compounds with desirable physicochemical and pharmacokinetic properties have been identified with activity against parasitic filarial nematodes. Two distinct chemical series were prioritized for preliminary structure activity relationship (SAR) evaluation. An *in vivo* filariasis study demonstrated macrofilaricidal POP activity for a compound from series A.

Bristol Myers Squibb (formerly Celgene Global Health), in collaboration with multiple global partners, has identified novel small molecules which act against a variety of parasitic filarial nematodes. Lead optimization efforts on series A were initiated along with continued *ex vivo* testing in subsequent *in vivo* murine and jird models. Current studies are ongoing and will be reported in due course.

## Declaration of competing interest

NAH, SC, and VK are former employees of BMS, GH is an employee of BMS and JZ is a former employee of Celgene. NAH, SC, VK, GH, and JZ are BMS shareholders with no other competing interest. All other authors have declared that no competing interests exist.

## Role of the funding source

Part of the work described in this paper was funded by former Celgene, currently 10.13039/100002491Bristol Myers Squibb (BMS) and 10.13039/100004774New England Biolabs. NAH, SC, and VK are former employees of BMS. GH is an employee of BMS. JZ is a former employee of Celgene. ZL and CC are employees of New England Biolabs. Work by SG, ST, AE, MK, N L-V, CM, AH, SS, and MH was funded by the 10.13039/501100014054Drugs for Neglected Diseases initiative through funding provided by 10.13039/100000200USAID. The Drugs for Neglected Diseases initiative (DNDi) is grateful to the following donors for funding part of this work: the 10.13039/100000865Bill & Melinda Gates Foundation, USA (Grant no. OPP1111431) and the US Agency For International Development (USAID). The contents are the responsibility of the Drugs for Neglected Diseases initiative and do not necessarily reflect the views of the donors. The funders had no role in the study design, data collection and analysis, decision to publish, or preparation of the manuscript.
