# Supplementary material for: Filarial nematode phenotypic screening cascade to identify compounds with anti-parasitic activity for drug discovery optimization
Source: Int J Parasitol Drugs Drug Resist. 2022 Jun 23;19:89–97. doi: 10.1016/j.ijpddr.2022.06.002 (PMC9278491; doi:10.1016/j.ijpddr.2022.06.002)
Supplement: Multimedia component 1 [file mmc1.docx]

**Supplementary Information**

**Supplementary** **Figure S1a** and **b** Immiticide (melarsomine used as a positive control) effect on adult *Onchocerca gutturosa* motility and MTT. Single experiment with two adult worms per condition. EC50s were calculated after 5 days of *in vitro* culture for the filarial motility and viability as assessed by the MTT assay.

**Supplementary** **Figure S2**. Immiticide (melarsomine used as a positive control) effect on against *Onchocerca lienalis* microfilaria. Single experiment with five microfilariae per condition. EC50s for the inhibition of microfilariae motility were calculated after 5 days of *in vitro* culture.

**Supplementary** **Figure S3**. Compounds 1 and 2 effect on adult *Onchocerca gutturosa* motility and MTT. Single experiment with two adult worms per condition. EC50s were calculated after 5 days of *in vitro* culture for the filarial motility and viability as assessed by the MTT assay.

**Supplementary** **Figure S4**. *Ex vivo* MTT worm viability screening against adult *O. gutturosa.* *O. gutturosa* adult worm viability was assessed using MTT/formazan colorimetry on day 5. Percentage reduction (compared to the untreated control) of formazan formation is correlated with worm damage or death at 12.5 µM. Single experiment with two adult worms per condition.

**Supplementary** **Figure S5**. Efficacy of candidate 1 and 2 in the *L. sigmodontis* mouse model of filariasis. Mice were infected with *L. sigmodontis* and treated at 33-35 days post infection orally three times per day with 30mg/kg of compound 2 (0.5% CMC/0.25% Tween 80 in Millipore water) for 5 days, and flubenzadole (2mg/kg, subcutaneously in 0.1% Tween 80 in 0.5% HEC once-daily for 5 days). Mice were euthanized at 75-78 days post infection and worms were counted. Single experiment with 5-7 animals per group.

**Supplementary Table S1**: *Ex vivo* screening against adult *O. gutturosa* and *O. lienalis* microfilariae, EC_50_ data, e*x vivo* MTT worm viability screening against reduction of motility, cellular toxicity in mammalian cells – monkey kidney cell feeder layer (LLCMK2, ECACC, UK) and *ex vivo B. malayi* and *L. sigmodontis* screening. Motility inhibition EC_50_ (µM) of *O. gutturosa* adult worms and *O. lienalis* microfilariae. Compounds were tested at a top concentration of 12.5µM and observed for reduction in motility as represented by the EC_50_ (µM) after five days of in vitro culture. Single experiment with two adult worms per condition**.** *O. gutturosa* adult worm viability was assessed using MTT/formazan colorimetry on day 5. *O. gutturosa* adult worm viability was assessed using MTT/formazan colorimetry on day 5. Percentage reduction (compared to the untreated control) of formazan formation is correlated with worm damage or death at 12.5 µM. Single experiment with two adult worms per condition. Cellular toxicity was evaluated using monkey kidney cell feeder layer (LLCMK2, ECACC, UK) used for the *O. lienalis* microfilariae assays The cell cultures are sub-passaged every 15-20 days using trypsin to detach the cells, and split in a 1:4 ratio. Single experiment with two adult worms per condition**.** Percent reduction in overall motility in adult *L. sigmodontis/B. malayi* filariae or *sigmodontis/B. malayi* microfilariae screened at 100nM. Shown are motility scores after 5 days of culture converted to a percentage reduction where a score of 4 (rapid movement and largely coiled) = 0% motility reduction and score of 0 (no motility, dead) = 100% motility reduction. Single experiment with 4-6 adult *L. sigmodontis/B. malayi* filariae or ~80 *L. sigmodontis/B. malayi* microfilariae per condition.

**Supplementary Table S2**: ***C. elegans* assessment of compound viability based on food consumption.** Compounds were tested at 50µM on L1 larvae of *C. elegans* in 96-well plates. Each well contained 100μL of S medium, 10 L1 larvae, 1µL of compound dissolved in DMSO or DMSO control, and dead E. coli. OD600nm was measured on day 1 and day 7 to measure the amount of E. coli inside the well. The difference in OD600nm between day 1 (E. coli added) and day 7 (E. coli remaining) was plotted. Single experiment with 10 larvae per condition. Worm development was examined microscopically daily using a standard dissecting microscope (Motic K series) and the worm condition (live/dead) was reported on day 7 of the culture. Viability was scored based on food consumption motility and morphology. Larval stage determination and development was assessed based on size and morphology as described. One generation of worm development was followed for growth defects/arrest. (Scoring: 0=Dead, 1=L1, 2=L1/2, 4=L2/3, 5=L3, 6=L3/4, 8=L4/Young Adult, 9=Young Adult/Adult, 10=Adult appears, 11=Adult with f1 Progeny)

**Supplementary Table S3**: **Analysis of chemical properties of series A-G.** Compounds were analyzed based upon their TPSA (total polar surface area), LogP, MW (molecular weight). Calculations (MW, TPSA, LogP, LE, LLE) were carried out and plotted using Dotmatics Vortex v2016.10.56814

**Supporting Information**

**Filarial Nematode Phenotypic Screening Cascade to Identify Compounds with Anti-Parasitic Activity for Drug Discovery Optimization**

**Supplementary** **Figure S1a** **and b** Immiticide (melarsomine used as a positive control) effect on against adult *Onchocerca gutturosa* motility and MTT. Single experiment with two adult worms per condition. EC_50_s were calculated after 5 days of *in vitro* culture for the filarial motility and viability as assessed by the MTT assay.

A


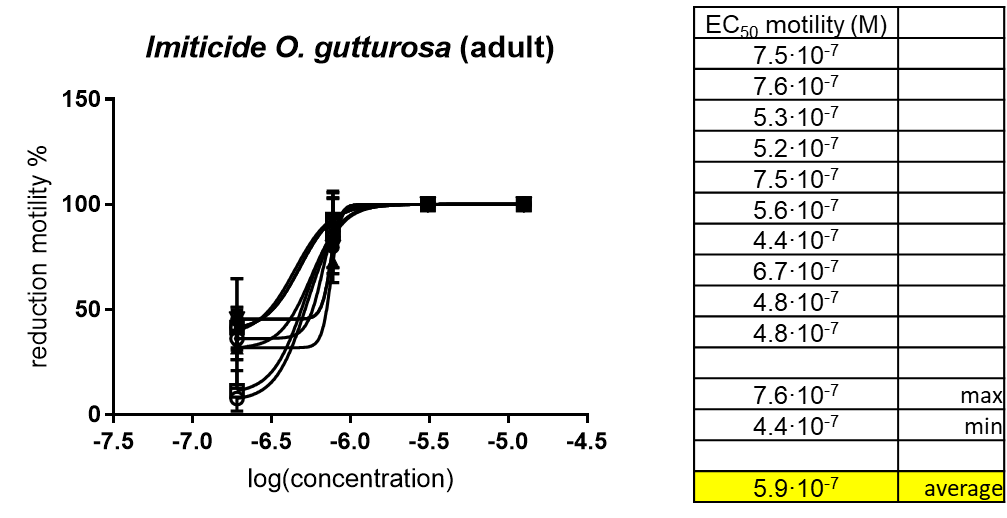


B


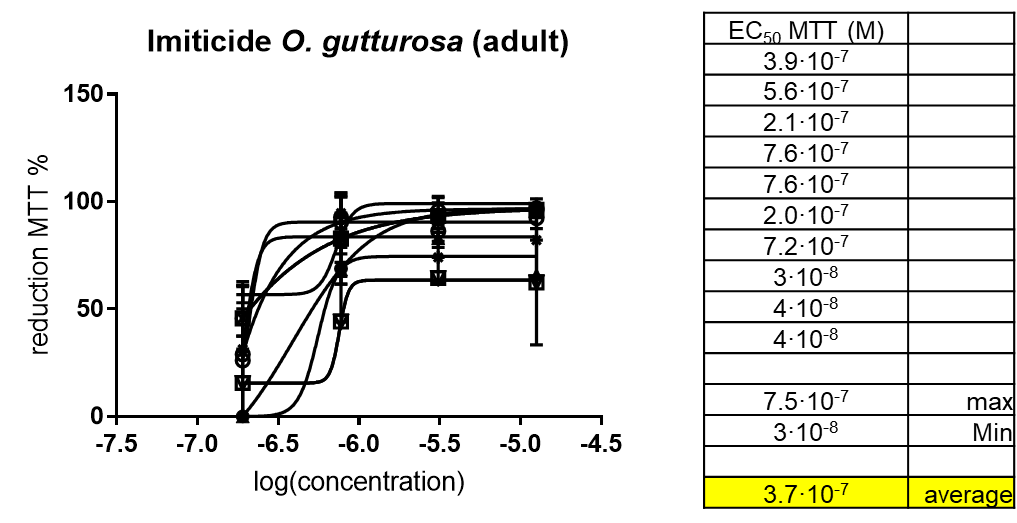


**Supplementary** **Figure S2**. Immiticide (melarsomine used as a positive control) effect on against *Onchocerca lienalis* microfilaria*.* Single experiment with five microfilariae per condition. EC_50_s for the inhibition of microfilariae motility were calculated after 5 days of *in vitro* culture.


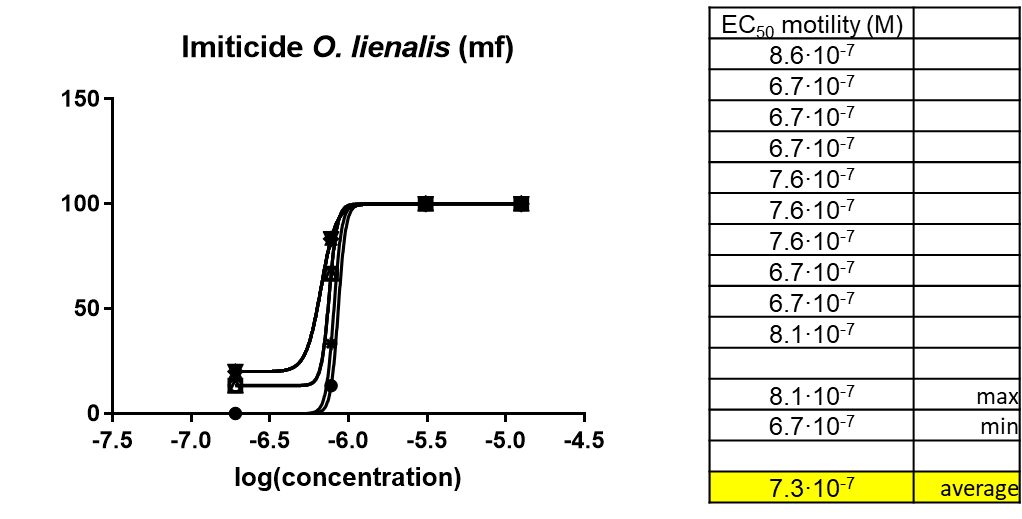


**Supplementary** **Figure S3**. Compounds 1 and 2 effect on against adult *Onchocerca gutturosa* motility and MTT. Single experiment with two adult worms per condition. EC_50_s were calculated after 5 days of *in vitro* culture for the filarial motility and viability as assessed by the MTT assay.


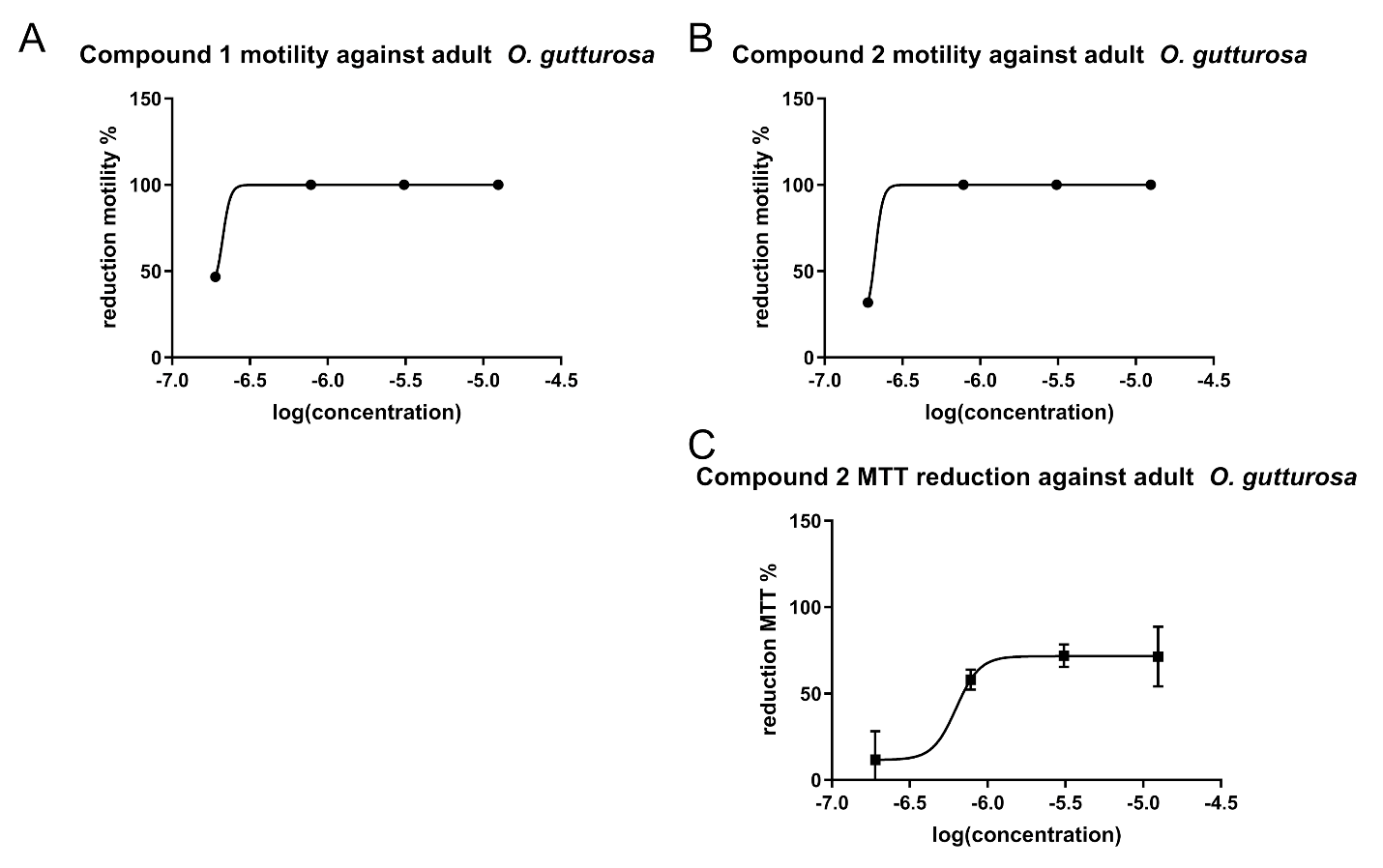


**Supplementary** **Figure S4**. ***Ex vivo* MTT worm viability screening against adult *O. gutturosa.*** *O. gutturosa* adult worm viability was assessed using MTT/formazan colorimetry on day 5. *O. gutturosa* adult worm viability was assessed using MTT/formazan colorimetry on day 5. Percentage reduction (compared to the untreated control) of formazan formation is correlated with worm damage or death at 12.5 µM. Single experiment with two adult worms per condition. Compound 1 is indicated by the green box, Compound 2 is indicated by the orange box.


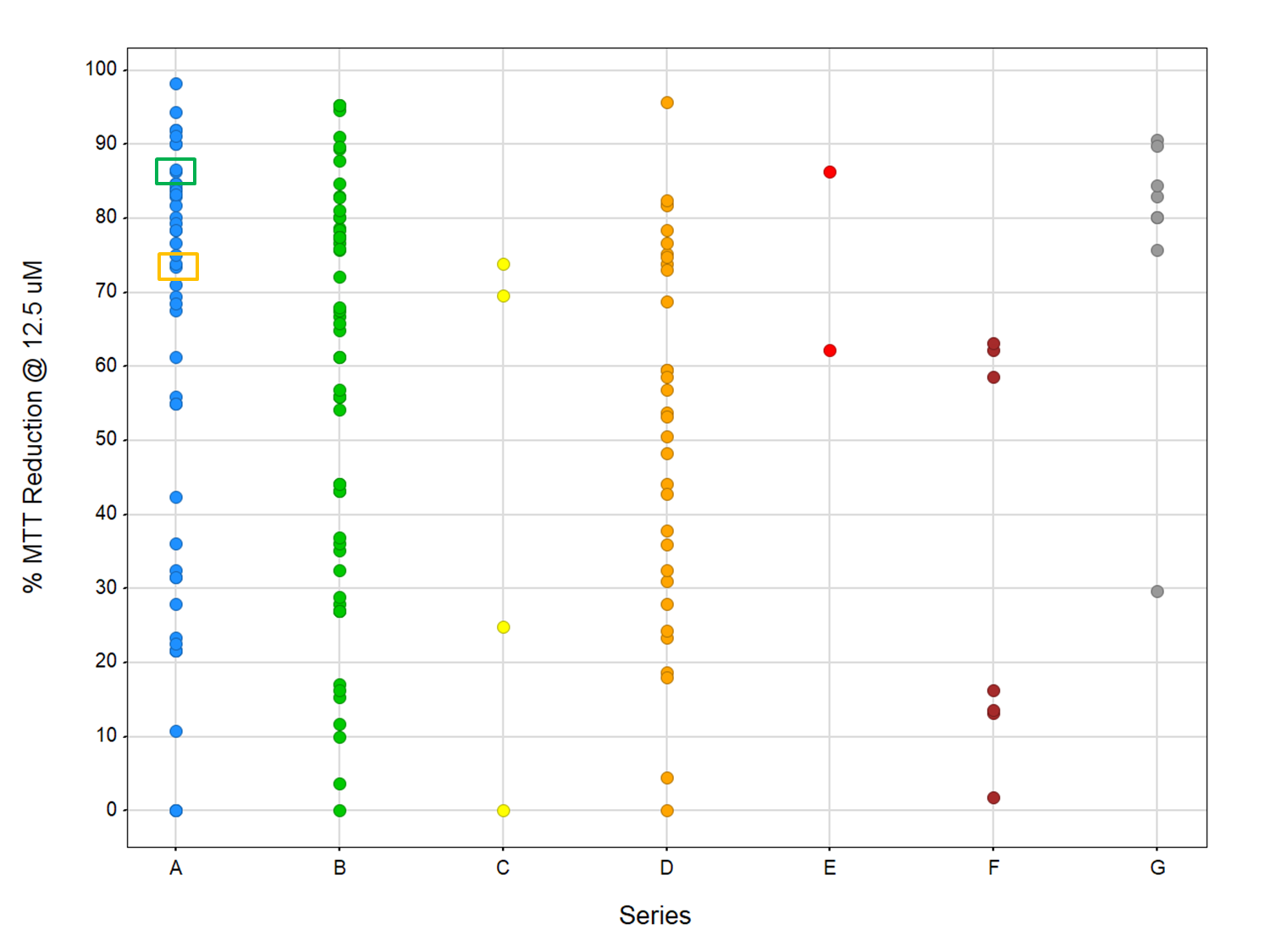


**Supplementary** **Figure S5**. Efficacy of candidat 1 and 2 in the *L. sigmodontis* mouse model of filariasis. Mice were infected with *L. sigmodontis* and treated at 33-35 days post infection orally three times per day with 30mg/kg of compound 2 (0.5% CMC/0.25% Tween 80 in Millipore water) for 5 days, and flubenzadole (2mg/kg, subcutaneously in 0.1% Tween 80 in 0.5% HEC once-daily for 5 days). Mice were euthanized at 75-78 days post infection and worms were counted. Single experiment with 5-7 animals per group.


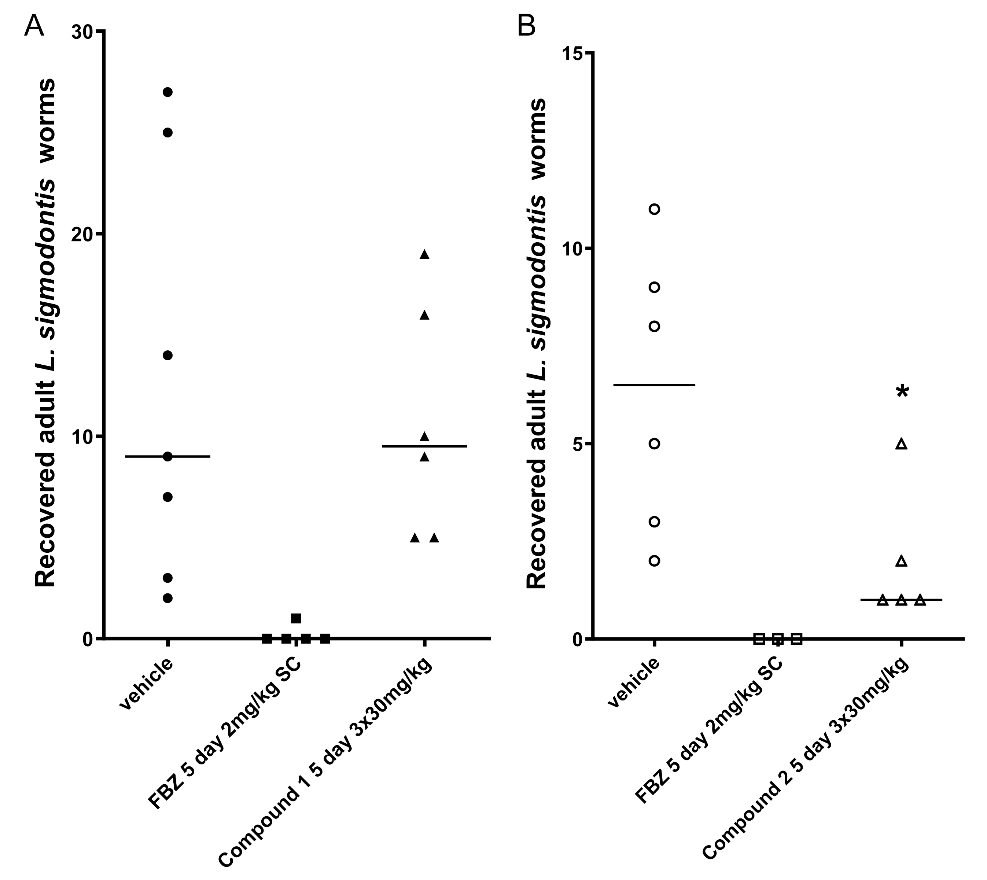


**Supplementary Table S1**: ***Ex vivo* screening against adult *O. gutturosa* and *O. lienalis* microfilariae, EC_50_ data, e*x vivo* MTT worm viability screening against reduction of motility, cellular toxicity in mammalian cells – monkey kidney cell feeder layer (LLCMK2, ECACC, UK) and *ex vivo B. malayi* and *L. sigmodontis* screening**  **.** Motility inhibition EC_50_ (µM) of *O. gutturosa* adult worms and *O. lienalis* microfilariae. Compounds were tested at a top concentration of 12.5µM and observed for reduction in motility as represented by the EC_50_ (µM) after five days of in vitro culture. Single experiment with two adult worms per condition**.** *O. gutturosa* adult worm viability was assessed using MTT/formazan colorimetry on day 5. *O. gutturosa* adult worm viability was assessed using MTT/formazan colorimetry on day 5. Percentage reduction (compared to the untreated control) of formazan formation is correlated with worm damage or death at 12.5 µM. Single experiment with two adult worms per condition. Cellular toxicity was evaluated using monkey kidney cell feeder layer (LLCMK2, ECACC, UK) used for the *O. lienalis* microfilariae assays The cell cultures are sub-passaged every 15-20 days using trypsin to detach the cells, and split in a 1:4 ratio. Single experiment with two adult worms per condition**.** Percent reduction in overall motility in adult *L. sigmodontis/B. malayi* filariae or *sigmodontis/B. malayi* microfilariae screened at 100nM. Shown are motility scores after 5 days of culture converted to a percentage reduction where a score of 4 (rapid movement and largely coiled) = 0% motility reduction and score of 0 (no motility, dead) = 100% motility reduction. Single experiment with 4-6 adult *L. sigmodontis/B. malayi* filariae or ~80 *L. sigmodontis/B. malayi* microfilariae per condition.

| Series | *O. g* MTT % Reduction @ 12.5 µM | *O. g* EC_50_ (µM) | *O. l* EC_50_ (uM) | Mammalian cells – monkey kidney cell feeder layer (LLCMK2, ECACC, UK) (µM) | *L. s* Adult motility % reduction at 100nM | *L. s* mf motility % reduction at 100nM | *B. m* Adult motility % reduction at 100nM | *B. m* mf motility % reduction at 100nM |
| --- | --- | --- | --- | --- | --- | --- | --- | --- |
| Imiticide (control) |  | 0.6 | 0.8 |  |  |  |  |  |
| A1 | 67.6 | 0.4 | 2.1 | >12.5 |  |  |  | 50.0 |
| A2 | 31.5 | >12.5 | >12.5 | >12.5 |  |  |  | 13.0 |
| A3 | 42.3 | >12.5 | >12.5 | >12.5 |  |  |  | 5.0 |
| A4 | 21.6 | >12.5 | >12.5 | >12.5 |  |  |  | 5.0 |
| A5 | 0 | >12.5 | >12.5 | >12.5 |  | 93.0 |  | 8.0 |
| A6 | 32.4 | >12.5 | >12.5 | >12.5 |  |  |  | 10.0 |
| A7 | 21.6 | >12.5 | >12.5 | >12.5 |  | 100.0 |  | 53.0 |
| A8 | 0 | >12.5 | >12.5 | >12.5 |  |  |  | 5.0 |
| A9 | 36 | >12.5 | >12.5 | >12.5 |  | 25.0 |  | 8.0 |
| A10 | 23.4 | >12.5 | >12.5 | >12.5 |  | 55.0 | 8.0 | 43.0 |
| A11 | 21.6 | >12.5 | >12.5 | >12.5 |  | 38.0 |  | 43.0 |
| A12 | 10.8 | >12.5 | >12.5 | >12.5 |  |  |  | 5.0 |
| A13 | 55.9 | >12.5 | >12.5 | >12.5 |  | 38.0 |  | 25.0 |
| A14 | 55 | >12.5 | >12.5 | >12.5 |  | 100.0 |  | 30.0 |
| A15 | 31.5 | >12.5 | >12.5 | >12.5 |  | 15.0 | 50.0 | 43.0 |
| A16 | 69.4 | 0.6 | 1.1 | 0.78 |  |  | 63.0 | 85.0 |
| A17 | 22.5 | >12.5 | >12.5 | >12.5 |  |  |  | 23.0 |
| A18 | 27.9 | >12.5 | >12.5 | >12.5 |  |  |  | 8.0 |
| A19 | 55 | >12.5 | >12.5 | >12.5 |  |  |  | 8.0 |
| A20 | 68.5 | >12.5 | >12.5 | >12.5 |  | 100.0 |  | 43.0 |
| A21 | 84 | 0.2 | >12.5 | 0.2 |  |  |  | 83.0 |
| A22 | 90.1 | 0.2 | >12.5 | >12.5 |  | 90.0 | 34.0 | 58.0 |
| A23 | 80.2 | 1.8 | >12.5 | >12.5 | 43 |  |  | 18.0 |
| A24 | 78.4 | >12.5 | >12.5 | >12.5 |  |  |  | 5.0 |
| A25 | 0 | >12.5 | >12.5 | >12.5 |  |  |  | 8.0 |
| A26 | 76.6 | >12.5 | >12.5 | >12.5 | 75 | 100.0 |  | 18.0 |
| A27 | 86.3 | 0.9 | >12.5 | >12.5 |  |  | 68.0 | 90.0 |
| A28 | 61.3 | >12.5 | 5.0 | >12.5 |  | 100.0 | 18.0 | 5.0 |
| A29 (cmpd 2) | 84.7 | 0.2 | 1.2 | >12.5 | 95 | 100.0 | 18.0 | 77.0 |
| A30 | 98.2 | 0.8 | >12.5 | >12.5 |  |  |  | 40.0 |
| A31 | 82.9 | >12.5 | >12.5 | >12.5 |  | 100.0 | 18.0 | 40.0 |
| A32 | 71 | 0.7 | >12.5 | >3.1 |  |  |  | 100.0 |
| A33 | 84.7 | 0.6 | >12.5 | >12.5 |  |  |  | 90.0 |
| A34 | 73.5 | 1.3 | >12.5 | >12.5 |  |  |  | 5.0 |
| A35 (cmpd 1) | 73.9 | 0.2 | 0.6 | >12.5 | 75.0 | 100.0 | 35.0 | 85.0 |
| A36 | 94.3 | 0.0041 | >12.5 | >12.5 |  |  |  | 35.0 |
| A37 | 83.8 | 0.8 | >12.5 | >3.1 |  |  | 95.0 | 8.0 |
| A38 | 91.9 | 0.9 | >12.5 | >12.5 |  |  |  | 10.0 |
| A39 | 81.7 | 0.6 | 0.9 | >3.1 |  |  |  | 98.0 |
| A40 | 79.3 | 0.2 | 4.1 | >3.1 | 100.0 | 100.0 |  | 93.0 |
| A41 | 91.9 | 0.2 | >12.5 | 0.78 |  | 100.0 | 100.0 | 93.0 |
| A42 | 90.1 | 0.7 | >12.5 | >12.5 |  |  |  | 15.0 |
| A43 | 83.2 | 0.8 | >12.5 | >12.5 |  |  | 25.0 | 8.0 |
| A44 | 91.1 | 0.6 | 0.8 | 0.2 |  | 100.0 | 100.0 | 100.0 |
| A45 | 78.4 | 0.9 | 3.1 | >3.1 | 57.0 | 100.0 | 88.0 | 45.0 |
| A46 | 75 | 0.3 | 0.9 | 0.78 |  | 100.0 | 45.0 | 90.0 |
| A47 | 86.5 | 0.2 | 0.5 | 0.2 |  |  | 100.0 | 100.0 |
| B1 | 35.1 | >12.5 | >12.5 | >12.5 |  | 100.0 | 5.0 | 68.0 |
| B2 | 17.1 | >12.5 | >12.5 | >12.5 |  |  |  | 0 |
| B3 | 43.2 | >12.5 | >12.5 | >12.5 |  | 100.0 |  | 28.0 |
| B4 | 15.3 | >12.5 | >12.5 | >12.5 |  |  |  | 10.0 |
| B5 | 44.1 | >12.5 | >12.5 | >12.5 |  |  |  | 8.0 |
| B6 | 66.7 | >12.5 | >12.5 | >12.5 |  |  |  | 8.0 |
| B7 | 3.6 | >12.5 | >12.5 | >12.5 |  |  |  | 8.0 |
| B8 | 11.7 | >12.5 | >12.5 | >12.5 |  | 13.0 |  | 8.0 |
| B9 | 27.9 | >12.5 | >12.5 | >12.5 |  |  |  | 10.0 |
| B10 | 16.2 | >12.5 | >12.5 | >12.5 |  | 98.0 | 5.0 | 25.0 |
| B11 | 43.2 | >12.5 | >12.5 | >12.5 |  |  |  | 8.0 |
| B12 | 55.9 | >12.5 | >12.5 | >12.5 |  | 100.0 |  | 8.0 |
| B13 | 78.6 | 0.3 | >12.5 | >3.1 |  |  |  | 80.0 |
| B14 | 61.3 | 2.4 | >12.5 | >12.5 |  |  | 88.0 | 65.0 |
| B15 | 27 | >12.5 | >12.5 | >12.5 |  |  |  | 8.0 |
| B16 | 0 | >12.5 | >12.5 | >12.5 |  |  | 15.0 | 8.0 |
| B17 | 36 | >12.5 | >12.5 | >12.5 |  | 100.0 |  | 8.0 |
| B18 | 76.6 | 0.3 | >12.5 | >12.5 |  |  |  |  |
| B19 | 72.1 | 1.3 | >12.5 | >12.5 |  |  |  |  |
| B20 | 64.9 | 1.4 | >12.5 | >12.5 |  |  |  |  |
| B21 | 27 | >12.5 | >12.5 | >12.5 |  | 100.0 | 24.0 | 5.0 |
| B22 | 55.9 | >12.5 | >12.5 | >12.5 |  | 100.0 | 16.0 | 35.0 |
| B23 | 84.7 | 0.9 | >12.5 | >12.5 |  |  |  | 5.0 |
| B24 | 87.8 | 1.0 | >12.5 | >12.5 | 36.0 | 100.0 |  | 25.0 |
| B25 | 78.4 | 0.7 | 4.95 | >3.1 |  |  |  | 10.0 |
| B26 | 55.9 | >12.5 | >12.5 | >12.5 |  |  |  | 5.0 |
| B27 | 54.1 | 0.9 | >12.5 | >12.5 |  | 100.0 | 30.0 | 0 |
| B28 | 61.3 | 0.7 | >12.5 | >12.5 | 30.0 | 100.0 | 58.0 | 40.0 |
| B29 | 80.2 | 0.6 | 4.1 | >3.1 |  |  |  | 5.0 |
| B30 | 32.4 | >12.5 | >12.5 | >12.5 |  |  |  | 3.0 |
| B31 | 77.5 | 0.2 | >12.5 | >12.5 |  |  |  | 3.0 |
| B32 | 67.6 | 0.2 | >12.5 | >12.5 | 100.0 | 100.0 | 43.0 | 30.0 |
| B33 | 67.6 | 0.7 | >12.5 | >12.5 |  |  |  | 20.0 |
| B34 | 27 | >12.5 | >12.5 | >12.5 |  | 100.0 | 18.0 | 8.0 |
| B35 | 9.9 | >12.5 | >12.5 | >12.5 |  |  | 43.0 | 5.0 |
| B36 | 89.4 | 0.5 | >12.5 | >12.5 |  |  |  | 10.0 |
| B37 | 94.6 | 0.9 | >12.5 | >12.5 | 28.0 | 100.0 | 68.0 | 20.0 |
| B38 | 82.9 | 0.7 | >12.5 | >12.5 |  | 100.0 |  | 25.0 |
| B39 | 95.3 | 0.2 | >12.5 | >12.5 |  |  | 100.0 | 55.0 |
| B40 | 75.7 | 0.9 | >12.5 | >12.5 |  |  |  | 5.0 |
| B41 | 77.5 | 0.2 | >12.5 | >12.5 |  | 100.0 | 75.0 | 83.0 |
| B42 | 80.2 | 0.2 | >12.5 | >12.5 |  |  |  | 5.0 |
| B43 | 68 | 1.0 | >12.5 | >12.5 | 58.0 |  | 100.0 | 93.0 |
| B44 | 77.5 | >12.5 | >12.5 | >12.5 |  |  |  | 8.0 |
| B45 | 28.8 | >12.5 | >12.5 | >12.5 |  |  |  | 8.0 |
| B46 | 91 | 0.4 | >12.5 | >12.5 | 92.0 | 100.0 | 30.0 | 20.0 |
| B47 | 89.6 | 0.1 | >12.5 | >12.5 | 78.0 | 93.0 | 2.0 | 5.0 |
| B48 | 81.1 | 2.1 | >12.5 | >12.5 |  |  | 38.0 | 5.0 |
| B49 | 75.8 | 0.8 | >12.5 | >12.5 | 18.0 |  |  | 5.0 |
| B50 | 65.8 | 2.6 | >12.5 | >12.5 | 13.0 | 100.0 | 38.0 | 23.0 |
| B51 | 36.9 | >12.5 | >12.5 | >12.5 |  |  |  | 5.0 |
| B52 | 77.5 | 0.2 | >12.5 | >12.5 |  |  |  | 8.0 |
| B53 | 82.8 | 1.2 | >12.5 | >12.5 | 13.0 |  | 55.0 | 8.0 |
| B54 | 95.3 | 0.2 | 0.9 | 0.78 | 68.0 |  |  | 53.0 |
| B55 | 44.1 | >12.5 | 1.1 | 0.78 |  | 100.0 |  | 5.0 |
| B56 | 56.8 | >12.5 | >12.5 | >12.5 |  |  |  | 5.0 |
| C1 | 24.8 | >12.5 | >12.5 | >12.5 |  | 18.0 |  | 35.0 |
| C2 | 0 | >12.5 | >12.5 | >12.5 |  | 18.0 | 13.0 | 78.0 |
| C3 | 69.5 | 0.2 | >12.5 | >12.5 |  |  |  |  |
| C4 | 73.9 | >12.5 | >12.5 | >12.5 |  |  | 18.0 |  |
| D1 | 53.8 | >12.5 | 2.6 | >12.5 |  |  |  | 0 |
| D2 | 75.2 | >12.5 | 1.7 | >12.5 |  |  |  | 0 |
| D3 | 31 | >12.5 | 3.6 | >3.1 |  |  |  | 0 |
| D4 | 76.6 | >12.5 | 5.0 | >12.5 |  |  |  | 0 |
| D5 | 18.6 | >12.5 | >12.5 | >12.5 |  |  |  |  |
| D6 | 81.9 | 0.8 | 3.0 | >12.5 |  |  |  | 0 |
| D7 | 59.5 | >12.5 | 1.6 | 0.78 |  |  |  | 0 |
| D8 | 27.9 | >12.5 | 3.3 | >3.1 |  |  |  | 0 |
| D9 | 95.7 | 0.2 | 3.4 | >12.5 |  |  |  | 0 |
| D10 | 73.9 | >12.5 | 2.8 | >12.5 |  |  |  | 0 |
| D11 | 37.8 | 12.5 | >12.5 | >12.5 |  |  |  | 0 |
| D12 | 68.7 | 2.0 | 1.0 | 0.78 |  |  |  | 93.0 |
| D13 | 50.5 | >12.5 | 2.8 | >12.5 |  |  |  | 100.0 |
| D14 | 23.4 | >12.5 | >12.5 | >12.5 |  |  |  | 0 |
| D15 | 81.7 | 1.3 | 2.8 | >12.5 |  |  |  | 100.0 |
| D16 | 82.4 | 2.0 | 3.3 | >3.1 |  |  |  | 100.0 |
| D17 | 53.2 | >12.5 | >12.5 | >12.5 |  |  |  | 5.0 |
| D18 | 44.1 | >12.5 | >12.5 | >12.5 |  |  |  | 0 |
| D19 | 56.8 | >12.5 | 2.4 | >12.5 |  |  |  | 100.0 |
| D20 | 0 | >12.5 | >12.5 | >12.5 |  |  |  |  |
| D21 | 18 | >12.5 | >12.5 | >12.5 |  |  |  | 0 |
| D22 | 48.3 | >12.5 | >12.5 | >12.5 |  |  |  | 0 |
| D23 | 42.8 | >12.5 | >12.5 | >12.5 |  |  |  | 0 |
| D24 | 32.4 | >12.5 | >12.5 | >12.5 |  |  |  | 10.0 |
| D25 | 35.9 | >12.5 | >12.5 | >3.1 |  |  |  | 0 |
| D26 | 4.5 | >12.5 | 2.8 | >12.5 |  |  |  |  |
| D27 | 59.5 | >12.5 | 2.6 | >12.5 |  |  |  |  |
| D28 | 74.8 | >12.5 | >12.5 | >12.5 |  |  |  |  |
| D29 | 58.6 | >12.5 | >12.5 | >12.5 |  |  |  |  |
| D30 | 73 | >12.5 | >12.5 | >12.5 |  |  |  |  |
| D31 | 24.3 | >12.5 | >12.5 | >12.5 |  |  |  |  |
| D32 | 78.4 | >12.5 | >12.5 | >12.5 |  |  |  |  |
| E1 | 62.2 | >12.5 | >12.5 | >12.5 |  | 10.0 |  |  |
| E2 | 86.3 | 9.2 | >12.5 | >12.5 |  | 15.0 | 38.0 | 0 |
| F1 | 13.1 | >12.5 | >12.5 | >12.5 |  | 13.0 |  | 15.0 |
| F2 | 58.6 | >12.5 | >12.5 | >12.5 |  | 35.0 | 43.0 | 0 |
| F3 | 1.8 | >12.5 | >12.5 | >12.5 |  | 13.0 |  | 23.0 |
| F4 | 13.5 | >12.5 | >12.5 | >12.5 |  | 5.0 |  | 43.0 |
| F5 | 62.2 | >12.5 | >12.5 | >12.5 |  | 5.0 |  | 0 |
| F6 | 63.1 | >12.5 | >12.5 | >12.5 |  | 5.0 |  | 0 |
| F7 | 16.2 | >12.5 | >12.5 | >12.5 |  | 7.5 |  | 0 |
| G1 | 82.9 | >12.5 | >12.5 | >12.5 |  |  |  |  |
| G2 | 84.4 | 0.9 | >12.5 | >12.5 |  |  |  |  |
| G3 | 29.7 | >12.5 | >12.5 | >12.5 |  |  |  | 0 |
| G4 | 80.2 | 0.6 | >12.5 | >12.5 |  |  |  |  |
| G5 | 80.2 | 3.8 | >12.5 | >12.5 |  |  |  | 0 |
| G6 | 75.7 | 3.0 | >12.5 | >12.5 |  |  |  |  |
| G7 | 90.6 | 0.7 | >12.5 | >12.5 |  |  |  |  |
| G8 | 89.8 | 0.6 | >12.5 | >12.5 |  |  |  |  |

**Supplementary Table S2**: ***C. elegans* assessment of compound viability based on food consumption.** Compounds were tested at 50µM on L1 larvae of *C. elegans* in 96-well plates. Each well contained 100μL of S medium, 10 L1 larvae, 1µL of compound dissolved in DMSO or DMSO control, and dead E. coli. OD600nm was measured on day 1 and day 7 to measure the amount of E. coli inside the well. The difference in OD600nm between day 1 (E. coli added) and day 7 (E. coli remaining) was plotted. Single experiment with 10 larvae per condition. Worm development was examined microscopically daily using a standard dissecting microscope (Motic K series) and the worm condition (live/dead) was reported on day 7 of the culture. Viability was scored based on food consumption motility and morphology. Larval stage determination and development was assessed based on size and morphology as described. One generation of worm development was followed for growth defects/arrest. (Scoring: 0=Dead, 1=L1, 2=L1/2, 4=L2/3, 5=L3, 6=L3/4, 8=L4/Young Adult, 9=Young Adult/Adult, 10=Adult appears, 11=Adult with f1 Progeny)

| Series | *C. elegans* Change in Food Consumption | *C. elegans* Phenotype |
| --- | --- | --- |
| Imiticide (control) |  |  |
| A1 | 0.703 | 11 |
| A2 | 0.593 | 11 |
| A3 | 0.67 | 11 |
| A4 | 0.679 | 11 |
| A5 | 0.731 | 11 |
| A6 | 0.691 | 11 |
| A7 | 0.309 | 11 |
| A8 | 0.682 | 11 |
| A9 | 0.713 | 11 |
| A10 | 0.738 | 11 |
| A11 | 0.744 | 11 |
| A12 | 0.619 | 11 |
| A13 | 0.616 | 11 |
| A14 | 0.635 | 11 |
| A15 | 0.081 | 11 |
| A16 | 0.136 | 0 |
| A17 | 0.625 | 11 |
| A18 | 0.296 | 11 |
| A19 | 0.544 | 11 |
| A20 | 0.551 | 11 |
| A21 | 0.121 | 9 |
| A22 | 0.107 | 11 |
| A23 | 0.708 | 11 |
| A24 | 0.467 | 11 |
| A25 | 0.061 | 11 |
| A26 | 0.598 | 11 |
| A27 | -0.023 | 11 |
| A28 | -0.027 | 11 |
| A29 (cmpd 2) | 0.315 | 11 |
| A30 | 0.688 | 11 |
| A31 | 0.111 | 0 |
| A32 | 0.083 | 8 |
| A33 | 0.671 | 11 |
| A34 | 0.658 | 11 |
| A35 (cmpd 1) | 0.418 | 11 |
| A36 | 0.636 | 11 |
| A37 | 0.18 | 1 |
| A38 | 0.553 | 11 |
| A39 | 0.176 | 11 |
| A40 | 0.659 | 11 |
| A41 | 0.535 | 11 |
| A42 | 0.213 | 11 |
| A43 | 0.663 | 11 |
| A44 | 0.081 | 11 |
| A45 | 0.663 | 11 |
| A46 | 0.83 | 9 |
| A47 | 0.665 | 11 |
| B1 | 0.686 | 11 |
| B2 | 0.641 | 11 |
| B3 | 0.685 | 11 |
| B4 | 0.726 | 11 |
| B5 | 0.703 | 11 |
| B6 | 0.679 | 11 |
| B7 | 0.661 | 11 |
| B8 | 0.577 | 11 |
| B9 | 0.65 | 11 |
| B10 | 0.662 | 11 |
| B11 | 0.633 | 11 |
| B12 | 0.645 | 11 |
| B13 | 0.195 | 11 |
| B14 | 0.107 | 11 |
| B15 | 0.651 | 11 |
| B16 | 0.763 | 11 |
| B17 | 0.666 | 11 |
| B18 | 0.642 | 11 |
| B19 | 0.636 | 11 |
| B20 | 0.623 | 11 |
| B21 | 0.558 | 11 |
| B22 | 0.636 | 11 |
| B23 | 0.677 | 11 |
| B24 | 0.653 | 11 |
| B25 | 0.745 | 11 |
| B26 | 0.663 | 11 |
| B27 | 0.177 | 8 |
| B28 | 0.603 | 11 |
| B29 | 0.641 | 11 |
| B30 | 0.644 | 11 |
| B31 | 0.662 | 11 |
| B32 | 0.663 | 11 |
| B33 | 0.712 | 11 |
| B34 | 0.597 | 11 |
| B35 | 0.265 | 5 |
| B36 | 0.646 | 11 |
| B37 | 0.639 | 11 |
| B38 | 0.665 | 11 |
| B39 | 0.171 | 0 |
| B40 | 0.669 | 11 |
| B41 | 0.691 | 11 |
| B42 | 0.644 | 11 |
| B43 | 0.699 | 11 |
| B44 | 0.651 | 11 |
| B45 | 0.622 | 11 |
| B46 | 0.688 | 11 |
| B47 | 0.609 | 11 |
| B48 | 0.636 | 11 |
| B49 | 0.644 | 11 |
| B50 | 0.669 | 11 |
| B51 | 0.745 | 11 |
| B52 | 0.68 | 11 |
| B53 | 0.722 | 11 |
| B54 | 0.15 | 2 |
| B55 | 0.226 | 4 |
| B56 | 0.728 | 11 |
| C1 | -0.051 | 8 |
| C2 | 0.11 | 8 |
| C3 | 0.786 | 11 |
| C4 | 0.522 | 11 |
| D1 | 0.605 | 11 |
| D2 | 0.322 | 11 |
| D3 | 0.528 | 11 |
| D4 | 0.494 | 11 |
| D5 | 0.642 | 11 |
| D6 | 0.097 | 6 |
| D7 | 0.104 | 8 |
| D8 | 0.284 | 11 |
| D9 | 0.104 | 10 |
| D10 | 0.065 | 11 |
| D11 | 0.65 | 11 |
| D12 | 0.083 | 11 |
| D13 | 0.433 | 11 |
| D14 | 0.375 | 11 |
| D15 | 0.223 | 11 |
| D16 | 0.056 | 10 |
| D17 | 0.616 | 11 |
| D18 | 0.649 | 11 |
| D19 | 0.064 | 10 |
| D20 | 0.738 | 11 |
| D21 | 0.593 | 11 |
| D22 | 0.673 | 11 |
| D23 | 0.092 | 5 |
| D24 | 0.262 | 11 |
| D25 | -0.155 | 11 |
| D26 | 0.612 | 11 |
| D27 | 0.739 | 11 |
| D28 | 0.717 | 11 |
| D29 | 0.799 | 11 |
| D30 | 0.812 | 11 |
| D31 | 0.626 | 11 |
| D32 | 0.178 | 6 |
| E1 | 0.126 | 9 |
| E2 | 0.044 | 8 |
| F1 | 0.012 | 11 |
| F2 | 0.454 | 11 |
| F3 | 0.651 | 11 |
| F4 | 0.099 | 6 |
| F5 | 0.121 | 4 |
| F6 | 0.166 | 1 |
| F7 | 0.099 | 0 |
| G1 | 0.451 | 11 |
| G2 | 0.584 | 11 |
| G3 | 0.458 | 11 |
| G4 | 0.512 | 11 |
| G5 | 0.633 | 11 |
| G6 | 0.7 | 11 |
| G7 | 0.57 | 11 |
| G8 | 0.548 | 11 |

**Supplementary Table S3**: **Analysis of chemical properties of series A-G.** Compounds were analyzed based upon their TPSA (total polar surface area), LogP, MW (molecular weight). Calculations (MW, TPSA, LogP, LE, LLE) were carried out and plotted using Dotmatics Vortex v2016.10.56814

| Series | MW | TPSA | LogP |
| --- | --- | --- | --- |
| Imiticide (control) |  |  |  |
| A1 | 255.3 | 91.8 | 2.3 |
| A2 | 252.7 | 70.2 | 4.0 |
| A3 | 304.4 | 140.5 | 4.0 |
| A4 | 303.4 | 133.2 | 3.7 |
| A5 | 290.4 | 70.2 | 4.5 |
| A6 | 280.8 | 70.2 | 4.6 |
| A7 | 290.4 | 70.2 | 4.8 |
| A8 | 292.3 | 79.5 | 3.5 |
| A9 | 252.7 | 70.2 | 4.0 |
| A10 | 329.3 | 73.5 | 4.3 |
| A11 | 313.8 | 73.5 | 4.0 |
| A12 | 305.4 | 100.7 | 2.2 |
| A13 | 253.4 | 73.5 | 3.1 |
| A14 | 296.4 | 96.0 | 2.2 |
| A15 | 247.4 | 66.1 | 4.6 |
| A16 | 254.3 | 78.9 | 2.8 |
| A17 | 283.4 | 86.3 | 4.1 |
| A18 | 285.3 | 66.1 | 4.6 |
| A19 | 335.4 | 66.1 | 5.4 |
| A20 | 259.4 | 66.1 | 3.9 |
| A21 | 284.3 | 88.2 | 2.7 |
| A22 | 245.3 | 66.1 | 3.5 |
| A23 | 217.3 | 66.1 | 2.6 |
| A24 | 254.3 | 78.9 | 2.4 |
| A25 | 331.4 | 108.6 | 3.1 |
| A26 | 269.3 | 76.1 | 3.3 |
| A27 | 322.3 | 78.9 | 3.8 |
| A28 | 317.4 | 108.6 | 2.8 |
| A29 (cmpd 2) | 284.3 | 88.2 | 2.7 |
| A30 | 269.3 | 91.8 | 2.7 |
| A31 | 274.4 | 107.2 | 4.0 |
| A32 | 282.4 | 78.9 | 3.3 |
| A33 | 282.4 | 78.9 | 3.3 |
| A34 | 298.4 | 88.2 | 3.4 |
| A35 (cmpd 1) | 298.4 | 88.2 | 3.0 |
| A36 | 298.4 | 88.2 | 3.0 |
| A37 | 364.4 | 78.9 | 4.9 |
| A38 | 257.3 | 94.7 | 3.2 |
| A39 | 296.4 | 78.9 | 3.8 |
| A40 | 282.4 | 78.9 | 3.3 |
| A41 | 298.4 | 88.2 | 3.0 |
| A42 | 293.4 | 94.7 | 3.4 |
| A43 | 311.4 | 122.0 | 2.1 |
| A44 | 322.4 | 78.9 | 4.5 |
| A45 | 384.5 | 106.6 | 3.2 |
| A46 | 342.4 | 97.4 | 3.6 |
| A47 | 312.4 | 88.2 | 3.4 |
| B1 | 267.4 | 82.8 | 3.5 |
| B2 | 254.4 | 80.6 | 2.0 |
| B3 | 226.3 | 83.6 | -0.1 |
| B4 | 287.3 | 71.9 | 2.6 |
| B5 | 288.3 | 84.7 | 1.5 |
| B6 | 287.3 | 71.9 | 2.6 |
| B7 | 287.3 | 71.9 | 2.5 |
| B8 | 287.3 | 71.9 | 2.5 |
| B9 | 279.3 | 85.1 | 2.5 |
| B10 | 310.8 | 84.5 | 2.4 |
| B11 | 225.3 | 54.5 | 1.8 |
| B12 | 239.3 | 54.5 | 2.3 |
| B13 | 302.3 | 67.4 | 2.7 |
| B14 | 318.8 | 67.4 | 3.2 |
| B15 | 312.4 | 67.4 | 3.0 |
| B16 | 238.3 | 80.9 | 0.8 |
| B17 | 300.3 | 80.9 | 2.4 |
| B18 | 290.4 | 96.5 | 3.4 |
| B19 | 270.4 | 96.5 | 3.1 |
| B20 | 290.4 | 96.5 | 3.3 |
| B21 | 327.4 | 110.5 | 1.7 |
| B22 | 328.3 | 104.7 | 1.8 |
| B23 | 330.4 | 67.4 | 2.9 |
| B24 | 248.3 | 67.4 | 1.5 |
| B25 | 274.3 | 80.9 | 2.5 |
| B26 | 274.3 | 96.1 | 1.9 |
| B27 | 285.3 | 80.3 | 1.7 |
| B28 | 288.3 | 85.3 | 1.2 |
| B29 | 288.3 | 85.3 | 1.7 |
| B30 | 298.4 | 67.4 | 2.7 |
| B31 | 309.3 | 91.2 | 2.3 |
| B32 | 312.4 | 67.4 | 3.1 |
| B33 | 353.2 | 67.4 | 3.8 |
| B34 | 356.4 | 105.5 | 1.6 |
| B35 | 275.3 | 109.0 | 1.4 |
| B36 | 285.3 | 80.3 | 1.3 |
| B37 | 288.3 | 85.3 | 1.7 |
| B38 | 288.3 | 80.9 | 2.7 |
| B39 | 288.3 | 85.3 | 2.2 |
| B40 | 288.3 | 85.3 | 2.2 |
| B41 | 312.4 | 67.4 | 3.0 |
| B42 | 262.3 | 67.4 | 1.8 |
| B43 | 309.3 | 91.2 | 2.3 |
| B44 | 397.5 | 88.7 | 3.0 |
| B45 | 353.4 | 79.5 | 3.1 |
| B46 | 309.3 | 91.2 | 2.3 |
| B47 | 292.3 | 80.9 | 2.6 |
| B48 | 292.3 | 96.1 | 2.0 |
| B49 | 385.4 | 148.6 | 0.4 |
| B50 | 359.4 | 108.6 | 1.3 |
| B51 | 304.3 | 105.3 | 1.8 |
| B52 | 388.5 | 105.5 | 1.6 |
| B53 | 412.5 | 123.0 | 1.1 |
| B54 | 302.4 | 85.3 | 2.5 |
| B55 | 274.3 | 96.1 | 1.7 |
| B56 | 356.4 | 104.7 | 2.0 |
| C1 | 319.3 | 70.3 | 6.9 |
| C2 | 350.4 | 84.7 | 3.4 |
| C3 | 391.5 | 93.2 | 3.3 |
| C4 | 416.5 | 84.7 | 5.2 |
| D1 | 340.5 | 59.1 | 3.4 |
| D2 | 360.9 | 59.1 | 3.6 |
| D3 | 344.4 | 59.1 | 3.1 |
| D4 | 360.9 | 59.1 | 3.6 |
| D5 | 356.5 | 68.3 | 2.9 |
| D6 | 332.4 | 49.8 | 5.6 |
| D7 | 352.4 | 49.8 | 5.5 |
| D8 | 292.4 | 49.8 | 4.8 |
| D9 | 332.4 | 49.8 | 5.5 |
| D10 | 304.4 | 49.8 | 4.9 |
| D11 | 293.4 | 62.7 | 2.9 |
| D12 | 335.5 | 62.7 | 3.8 |
| D13 | 333.4 | 62.7 | 3.9 |
| D14 | 361.5 | 91.0 | 3.8 |
| D15 | 347.5 | 62.7 | 4.2 |
| D16 | 355.4 | 62.7 | 4.3 |
| D17 | 321.4 | 62.7 | 3.6 |
| D18 | 307.4 | 62.7 | 3.4 |
| D19 | 347.5 | 62.7 | 4.2 |
| D20 | 336.4 | 91.8 | 1.6 |
| D21 | 319.4 | 62.7 | 3.5 |
| D22 | 341.4 | 62.7 | 4.0 |
| D23 | 338.5 | 78.1 | 5.7 |
| D24 | 407.6 | 81.3 | 5.5 |
| D25 | 302.4 | 78.1 | 4.8 |
| D26 | 350.4 | 68.3 | 3.8 |
| D27 | 350.4 | 68.3 | 3.8 |
| D28 | 334.4 | 63.3 | 4.6 |
| D29 | 350.8 | 63.3 | 5.1 |
| D30 | 338.4 | 59.1 | 4.0 |
| D31 | 328.8 | 70.1 | 3.5 |
| D32 | 356.8 | 106.8 | 5.7 |
| E1 | 318.7 | 78.5 | 3.4 |
| E2 | 318.7 | 78.5 | 3.4 |
| F1 | 391.4 | 63.5 | 5.0 |
| F2 | 337.3 | 54.2 | 4.5 |
| F3 | 288.3 | 84.7 | 1.2 |
| F4 | 345.8 | 58.9 | 5.5 |
| F5 | 345.8 | 58.9 | 5.5 |
| F6 | 345.8 | 58.9 | 5.0 |
| F7 | 345.8 | 58.9 | 5.0 |
| G1 | 399.9 | 79.7 | 4.9 |
| G2 | 385.9 | 79.7 | 4.5 |
| G3 | 399.9 | 79.7 | 4.9 |
| G4 | 414.0 | 93.7 | 5.4 |
| G5 | 428.0 | 93.7 | 5.6 |
| G6 | 428.0 | 93.7 | 5.6 |
| G7 | 399.9 | 93.7 | 4.9 |
| G8 | 399.9 | 93.7 | 4.9 |
